# Tuning the pH of Activation of Fluorinated Hydrazone-Based Switches—A Pathway to Versatile ^19^F Magnetic Resonance Imaging Contrast Agents

**DOI:** 10.1021/acssensors.2c02251

**Published:** 2023-01-25

**Authors:** Dawid Janasik, Patrycja Imielska, Tomasz Krawczyk

**Affiliations:** Department of Chemical Organic Technology and Petrochemistry, Silesian University of Technology, Krzywoustego 4, 44-100Gliwice, Poland

**Keywords:** molecular switches, 19F MRI, pH, hydrazone, substituents, structure−property relationship

## Abstract

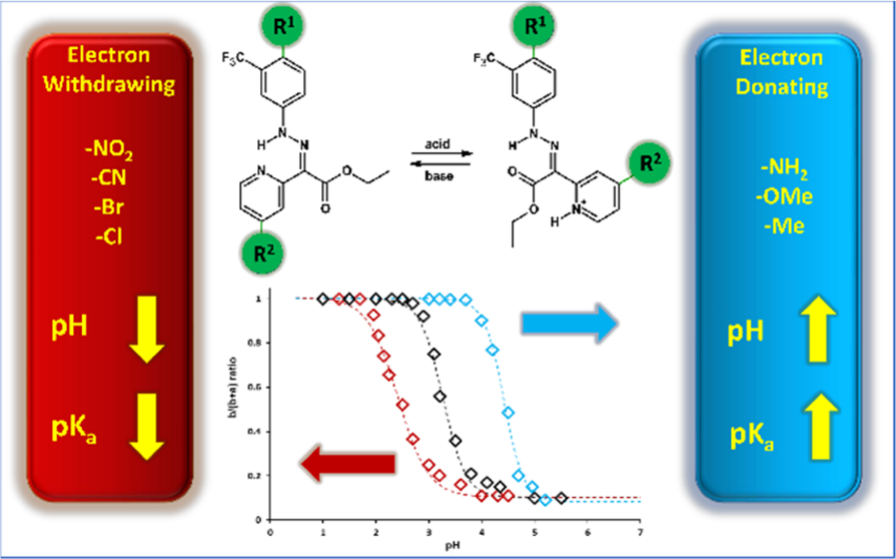

Molecular switches have become an area of great interest in recent years. They are explored as high-density data storage and organic diodes in molecular electronics as well as chemosensors due to their ability to undergo a transition between well-defined structures under the action of external stimuli. One of the types of such switches is hydrazones. They work by changing the configuration from E to Z under the influence of pH or light. The change in configuration is accompanied by a change in the absorption band and changes in the nuclear magnetic resonance (NMR) spectrum. In this publication, the structure–property relationship of fluorinated hydrazone switches was established. A linear relationship between the Hammett substituent constants and the pH where the switching occurs was found. Introduction of strong electron-donating groups allowed obtaining a hydrazone switch of p*K*_a_ = 6 suitable for application in ^19^F MRI as contrast agents.

Measurements of pH are important in many areas of chemical research as well as in agriculture,^1^ food industry,^2^ microbiology,^3^ environmental sciences,^4^ and in medicine.^5^ The pH value is typically measured using colorimetric indicators such as methyl orange or phenolphthalein^6^ or with more accurate potentiometric methods.^7^ In medical diagnostics, besides classical chemical methods, imaging techniques such as positron emission tomography (PET) and magnetic resonance imaging (MRI) are also applied for pH measurements.^8,9^

MRI is a noninvasive and widely used technique for imaging soft tissues with a spatial resolution of approximately 1 mm.^9−11^ It takes advantage of the magnetic properties of ^1^H nuclei of ubiquitous water molecules.^12,13^ Another, complementary, approach is the use of the resonance of ^19^F nuclei. Because fluorine is not common in organisms except for bones and teeth, only ^19^F nuclei introduced with a contrast agent can be observed.^14,15^ This offers a negligible background signal and high selectivity compared to the ^1^H MRI technique.^16−19^ On the other hand, high concentrations of fluorinated contrast agents are required, usually in the 1–10 mM^9^ range compared with traditional ^1^H contrast agents where only 0.05–0.1 mM are needed.^10^ The method is extensively researched with numerous ingenious inventions that open up newer and newer possibilities and bring the technique closer to medical applications.^9^ The most promising area of ^19^F MRI research is so-called intelligent or smart contrast agents,^20−22^ offering the possibility of imaging of enzymatic activity,^23^ the presence of specific ions,^24^ temperature,^25^ oxygenation,^26^ or pH.^22,27^ The latter is mostly used for imaging tumors based on the differences in acidity (pH 5–7) compared with healthy tissues (pH 7.4).^28,29^

Many pH-sensitive agents have been proposed for ^19^F MRI such as PEGylated nanogels containing perfluorocarbons,^30^ fluorinated pyridoxine,^31^ C_6_F_6_-loaded Au-fluorescent mesoporous silica nanoparticles,^32^ or copolymers^33−35^ that rely on the volume phase transition, decomposition, or isomerization as mechanisms of signal activation. Recently, we proposed molecular switches as potential ^19^F MRI agents.^36^ The main advantage is that no external reference either for chemical shift^31^ or for concentration^33^ is necessary, as they act as ratiometric probes not affected by differences in the pharmacokinetic properties of a probe and a reference.

A molecular switch is defined as a molecule capable of reversibly shifting between two (or more) thermodynamically stable states.^37−40^ The main advantage of molecular switches is the possibility of precise control of their properties using external stimuli. There are several classes of molecular switches, including photochromic, host–guest, rotaxanes, and hydrazones.^38^

Hydrazones as molecular switches were proposed in 2009 by Aprahamian.^41^ The use of the hydrazone moiety in conjunction with the pyridyl and ester groups allowed facile E/Z isomerization due to rotation about a N=C hydrazone bond in response to pH changes or other stimuli.

As a result of the configuration change, the hydrazone molecular switch changes its absorption band, ^1^H NMR, and, if a fluoroorganic group is appropriately introduced, also ^19^F NMR spectrum,^36^ which facilitates ^19^F MR imaging of the pH gradient. In the case of a native hydrazone switch bearing a single CF_3_ or F functional group, the switching process takes place in the pH range of 3–4,^9^ which is adequate for the gastric environment^42^ but insufficient for tumor imaging (5.0–6.5).^28^

Earlier work by the Aprahamian group indicated that the substitution of the aniline aromatic ring of a hydrazone switch affects the strength of the N–H···N hydrogen bond.^43,44^ Essentially, its strength was lowered with the electron-donating effect of the substituent, leading to the decreased p*K*_a_ of hydrazones. However, the extent to which the pH range of isomerization can be shifted by substitution of either a pyridine ring or an aniline ring was unclear. This question prompted us to investigate how hydrazone-based switches should be modified to precisely tune the pH of the transition process to the physiologically relevant pH range of 6–7.

For this purpose, a series of hydrazone switches containing a single −CF_3_ moiety as well as electron-donating (EDG) or electron-withdrawing (EWG) groups in both aromatic rings of a hydrazone was obtained to establish the structure–property relationship and to identify the structure of a switch suitable for pH imaging at the desired range.

## Experimental Section

### Synthesis

All reagents and starting materials were purchased from commercial vendors and used without further purification. All experiments were conducted in the air unless otherwise noted. Detailed NMR and mass spectrometry (MS) results for the compounds are provided in the Supporting Information.

Procedure 1. Ethyl 2-(pyridin-2-yl)acetate derivatives were synthesized following the literature procedure:^36^*n*-BuLi (2.5 M solution in hexanes, 2.05 equiv) was added dropwise to a stirred solution of diisopropylamine (2.10 equiv) in THF at −78 °C under Ar. The resulting solution was warmed for 1 h at room temperature to 0 °C and stirred at 0 °C for 1 h. Then, the solution was transferred to a stirred solution of modified 2-picoline (1.0 equiv) and diethyl carbonate (3.0 equiv) in THF at −78 °C under Ar. The resulting solution was stirred at −78 °C for 1 h, then allowed to warm while stirring to room temperature (30 min), and stirred for 1 h at room temperature. Then, 10 mL of a saturated aqueous solution of NH_4_Cl and 50 mL of water were added. The aqueous layer was extracted (3 × 15 mL) with Et_2_O. The organic layer was combined with the ether extracts, dried with MgSO_4_, and evaporated under reduced pressure to give the product (yield 70–85%) as a bright yellow oil.

**Figure 1:**
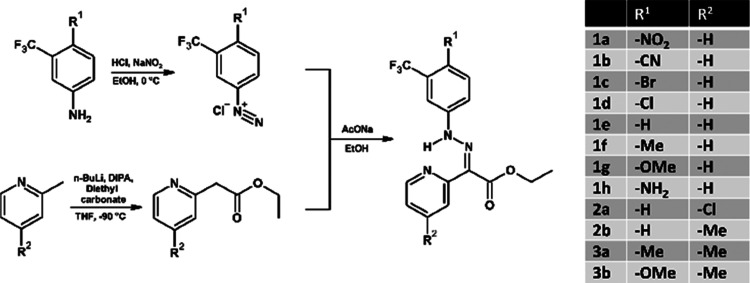
Synthesis scheme of fluorinated hydrazone molecular switches. Reaction conditions: (i) HNO_2_, ethanol, HCl_(aq.)_, 0 °C, 1 h; (ii) *n*-BuLi, DIPA, THF, −90 °C, 3 h; (iii) ethanol/water, AcONa, rt, 12 h.

Procedure 2. Hydrazone molecular switches (1a–1g, 2a, 2b, 3a, 3b, 4a, Figure 1) were obtained according to the modified literature procedure.^36^ Trifluoromethylaniline (1 equiv) was dissolved in a mixture (1:5 ratio) of 38% HCl and anhydrous (99.9%) EtOH and stirred in an ice bath for 30 min. A cold (≈0 °C) aqueous solution of NaNO_2_ (1 equiv) was then added dropwise over a period of 30 min. The obtained solution of diazonium salt was then added dropwise to a suspension of ethyl 2-pyridylacetate (1 equiv) and sodium acetate (6.4 equiv) in a cooled (0 °C) ethanol/water (8:1) mixture. The reaction mixture was stirred overnight and then washed with methylene chloride. The organic fraction was washed twice with saturated sodium bicarbonate solution and dried over MgSO_4_. The crude product was then subjected to silica gel column chromatography (hexane/EtOAc 90:10–60:40 depending on the compound) to give pure products (yield 30–70%).

Procedure 3. 1a/4a was dissolved in ethanol, and a tin(II) chloride/ethanol suspension was added. The mixture was heated at 50 °C for 2 h. The solvent was then removed under reduced pressure, and sodium hydroxide solution was then added. The resulting mixture was steam-distilled. The suspension was extracted with chloroform. The organic layer was dried with MgSO_4_ and then evaporated to dryness on a rotary evaporator. The residue was crystallized from Et_2_O and then recrystallized twice from the same solvent to obtain a red crystalline product (yield = 78%).

### Instrumental Measurements

High-resolution-mass spectrometry (HR-MS) was performed with ultra-performance liquid chromatography-MS (UPLC-MS) (Waters Xevo G2 QTof, ESI ionization, TOF detection). NMR spectra were recorded on a 400 MHz Agilent spectrometer and referenced internally using the residual protonated solvent resonances relative to tetramethyl silane (δ = 0 ppm) or trifluoroacetic acid (^19^F NMR, δ = −76.5 ppm). Ultraviolet–visible (UV–vis) spectra were recorded using a JASCO V-650 UV–vis spectrophotometer. A pHenomenal MD 8000L pH meter with a standard glass electrode was used for the pH measurements. A 3 M KCl solution was used as a reference to pH = 7.

### Computational Methods

Density functional theory (DFT) calculations were carried out using a B3LYP hybrid functional combined with a 6–31G (d, p) basis set, CPCM (acetonitrile). All calculations were performed using Orca 4.1.1 software. Statistical calculations were performed in Microsoft Excel.

### Switching Measurements

Solutions (5 mM) of molecular switches were prepared in acetonitrile/water (1:1) with the addition of 50 μL of D_2_O and 10 μL of fluorobenzene as an internal standard. Aqueous solutions of hydrochloric acid, nitric acid, trifluoroacetic acid, and acetic acid of concentrations from 0.5 mM to 1 M were used to induce the switching process, which was monitored by means of ^1^H, ^19^F NMR, or UV–vis.

## Results and Discussion

Three series of compounds of the general structure presented in Figure 1 were obtained. The −CF_3_ group was located in the meta position of the phenyl ring. This was due to the higher commercial availability of substituted anilines and the minimal impact of switching on the chemical shift of fluorine. Compounds 1a–1h contained a substituent in the phenyl ring, 2a and 2b in the pyridine ring, while 3a–3b contained substituents in both rings. This allowed the evaluation of the impact of the substitution on the p*K*_a_ of molecular switches depending on the Hammett constant of the substituents and their positions. Series 1 contained eight different compounds with substituents of ascending electron-donating nature starting with the −NO_2_ group and ending with the −NH_2_ group. Series 2 was modified in the pyridine ring by −Cl and −Me groups. Series 3 was modified in both rings yielding substituents with the −Me or −OMe group.

**Figure 2:**
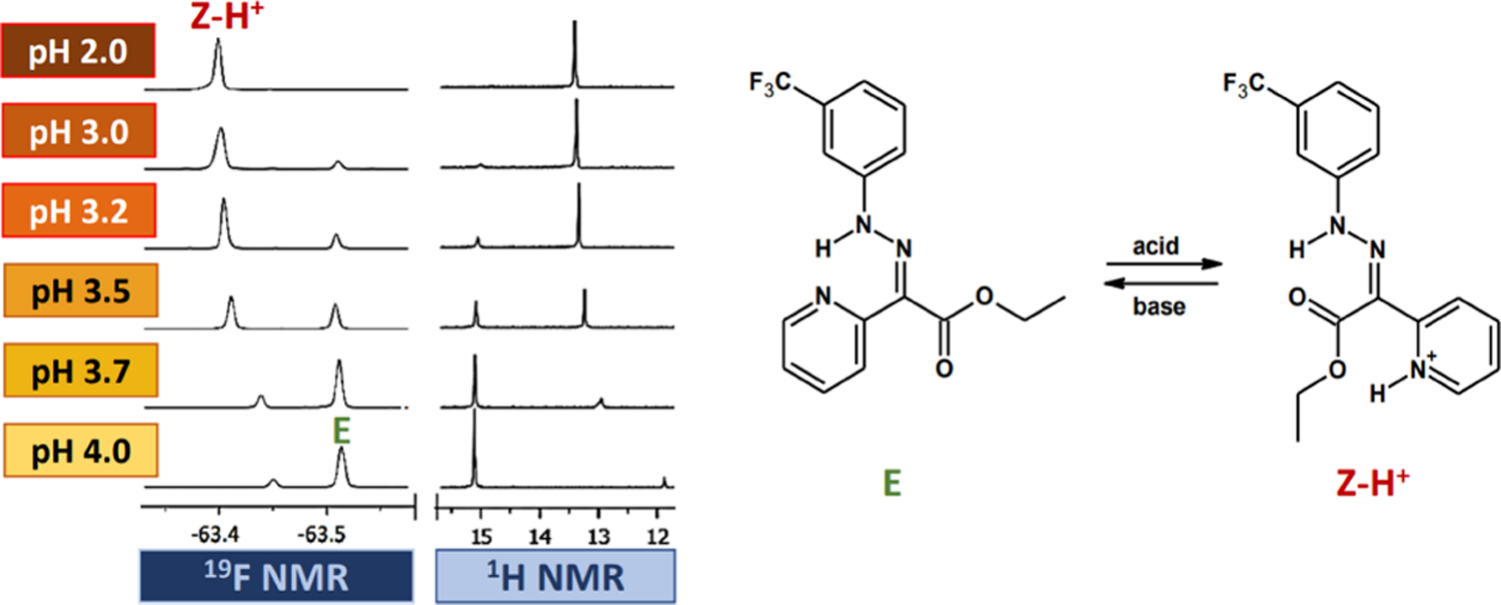
Hydrazone switching mechanism under the influence of acid and base, with observations of ^1^H and ^19^F NMR spectra.

The mechanism of the switching process (Figure 2) is based on the protonation of the pyridine subunit, which forces E/Z isomerization. The process is manifested by changes in the ^1^H NMR and ^19^F NMR spectra, and a color change in the solution (usually from orange to yellow, Figure S28). In the case of ^19^F NMR spectra, peaks of the −CF_3_ group can be seen near −63.5 ppm before and −63.4 ppm after the addition of an acid. At ^1^H NMR spectra, the changes are more pronounced with the hydrazone N–H proton visible at 15 ppm replaced with a new signal at 13 ppm. This shift indicates that rotation around the C=N bond has occurred (E/Z isomerization), in addition to the formation of a hydrogen bond by the N–H proton with the carbonyl group of the ester subunit, yielding Z–H^+^.

**Figure 3:**
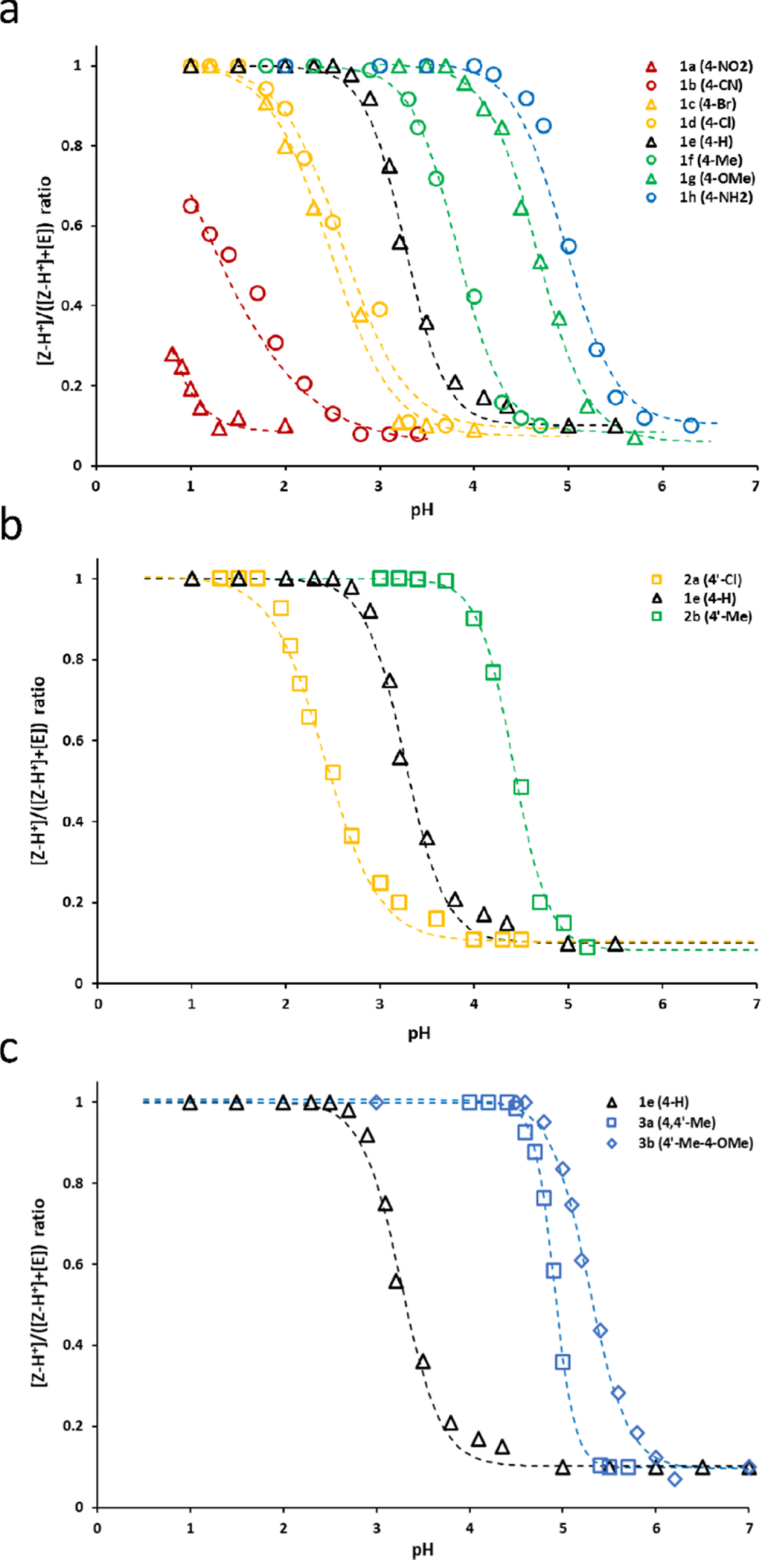
^19^F NMR titration curves of series 1 (a), 2 (b), and 3 (c). Peak notation as in Figure 2. Sigmoidal curves were fitted to the data using the Microsoft Excel Solver add-in and the least-squares method.

To precisely determine the p*K*_a_ of molecular switches, titration experiments were performed where the progress of isomerization was monitored by ^19^F NMR (Figure 3). The ratio of the area under the Z–H^+^ peak (protonated form) to the sum of the areas under peaks corresponding to E and Z–H^+^ was used to quantify the progress of the switching process.

**Figure 4:**
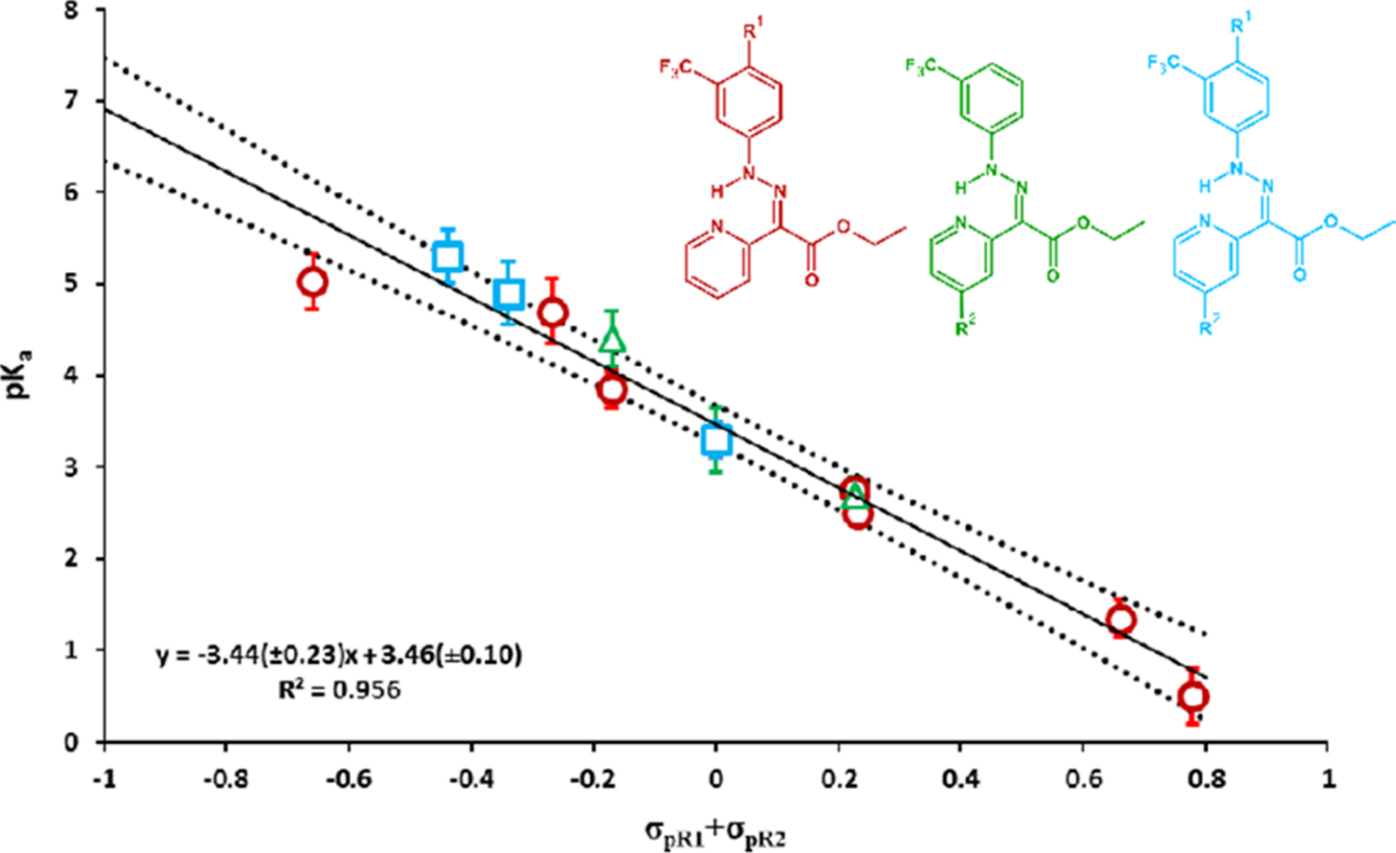
Relationship between substituents’ Hammett constants and the p*K*_a_ of hydrazone switches in series 1–3. The dashed lines represents the confidence interval of linear regression at α = 0.05.

The pK_a_ of each switch was calculated from regression data assuming p*K*_a_ = pH, where [Z–H^+^]/([Z–H^+^]+[E]) = 0.5. The results were plotted against the sum of *R*_1_ and *R*_2_ Hammett substituent constants introduced to aniline and pyridine rings (Figure 4). The relationship was linear with only minor differences among series 1–3. If each series was considered separately, the slopes were higher if substituents were introduced to the pyridyl ring (−4.6 and −4.2 for series 2 and 3 respectively, not statistically different) than series 1 (−3.2), where only the aniline ring was substituted (Figure S27).

**Figure 5:**
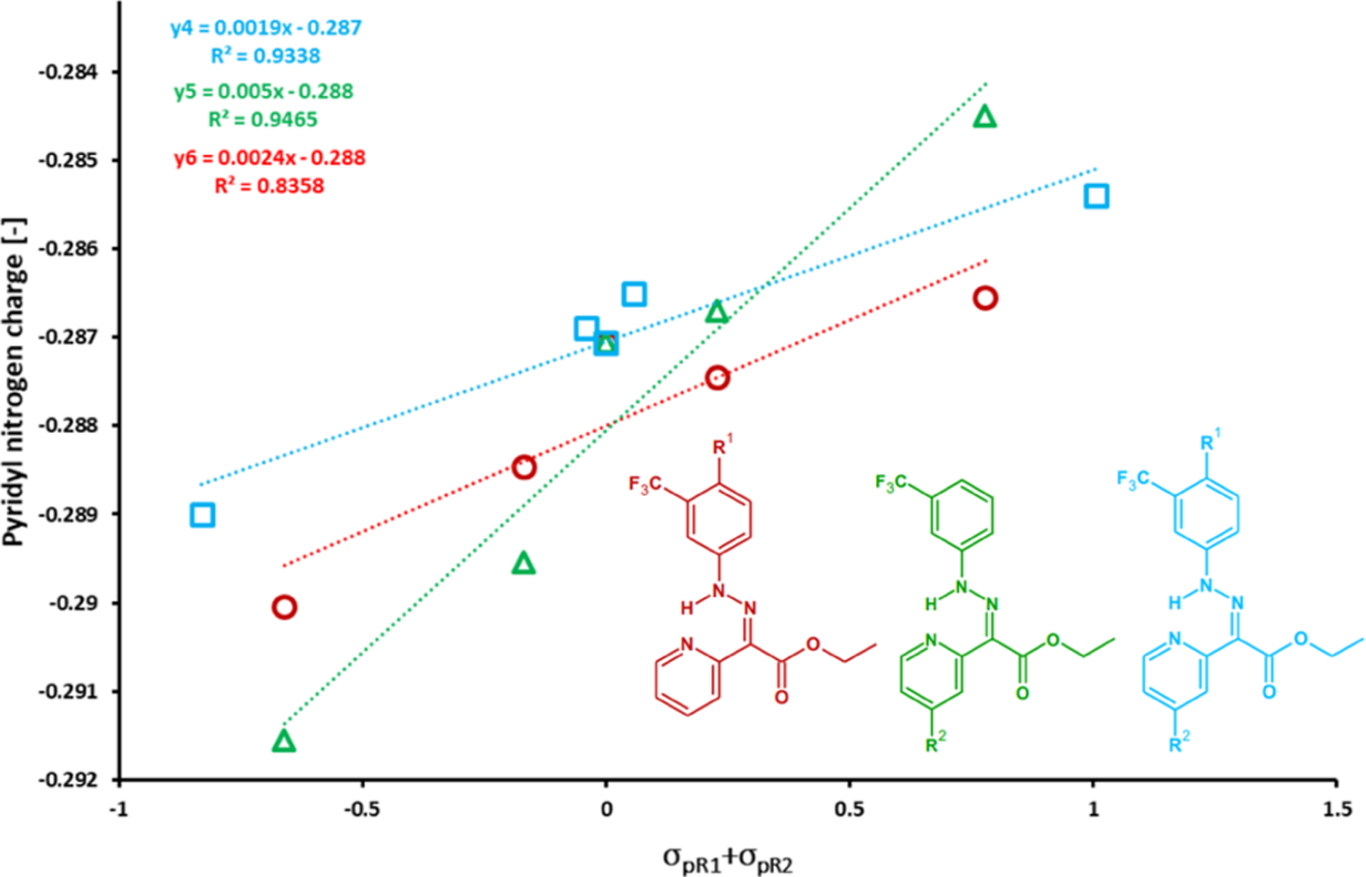
DFT-calculated partial negative charge of pyridyl nitrogen in hydrazone switches depending on the position and Hammett constant of the substituents.

The observed relationship and apparent differences among series 1–3 can be explained by the effect of substitution of pyridyl and phenyl rings on the basicity of pyridyl nitrogen. In both cases, the partial negative charge on the pyridyl nitrogen increases with the electron-donating strength of the substituents (regardless of their position), due to possible resonance between both rings through the HN–N=C–C=N fragment. The effect can be quantified with DFT calculations, showing that the negative charge on the pyridyl nitrogen increases with the donor strength of the substituent.^43,45,46^ If the substituent is positioned in the pyridine ring, the charge changes more rapidly with the substituent’s Hammett constant than that with substitution of phenyl or both rings. The effect is evident from the respective slopes of linear regression lines 0.005 (green) versus −0.0024 (red) and 0.0019 (blue; Figure 5).

Additionally, we assessed the effect of field-inductive (σ_F_) and resonance (σ_R_) constants of the substituents on the p*K*_a_ of the switches.^47^ For series 1, the resonance effect was 60% stronger than the inductive effect (p*K*_a_= −4.5σ_R_ −2.8σ_F_ +3.3), while for series 2, both effects were comparable (p*K*_a_= −4.0σ_R_ −3.5σ_F_ +3.6).

Based on the general relationship between the substituent effect and the p*K*_a_ of hydrazones (Figure 4), we attempted to obtain a switch of p*K*_a_ > 6 suitable for the ^19^F MRI of tumors. Based on the slope and the intercept, the sum of the Hammett constant should be at least −0.59. Considering the commercial availability of the reagents, a hydrazone switch with the −NH_2_ group in phenyl and the −CH_3_ group in the pyridine ring 4b was obtained. Additionally, we decided to change the position of the −CF_3_ group from para to ortho to increase the difference in chemical shifts between the E and Z–H^+^ states. Such a change should have minimal effect on the p*K*_a_ of the resulting switch because of only 0.1 differences in Hammett constants of o-CF_3_ and p-CF_3_ groups and nearly identical p*K*_a_ of CF_3_-substituted hydrazone switches regardless of the position of the −CF_3_ group.^36^

**Figure 6:**
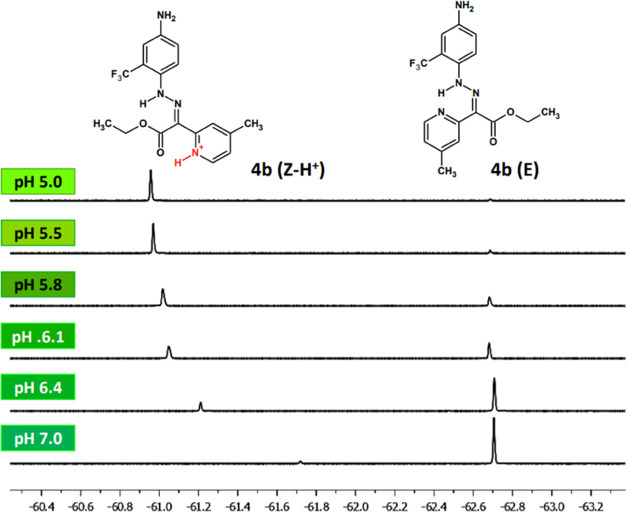
Titration of 4b in water by 0.05 mM acetic acid followed by ^19^F NMR.

The synthetic procedure was identical for compound 1h (procedure 3) with only 1a replaced by 4a. The resulting switch 4b was soluble in water. The switching process took place at pH from 5.5 to 6.4 with a calculated p*K*_a_ of 6.05 (Figure 6). We observed no signs of degradation of the probe even after 1 week at pH < 3. The titration of 4b with 0.05 mM acetic acid in aqueous solution showed a 1.8 ppm difference in the chemical shift between E and Z–H^+^ isomers at the ^19^F NMR spectrum (Figure 6). The corresponding peak widths at half height were 9 Hz in both cases. This difference facilitates the potential use of 4b in ^19^F MRI, as a 0.5 ppm difference can be considered minimal to allow a short acquisition time.^36^ No changes in transverse and longitudinal relaxation times (*T*_1_ ≈ *T*_2_ ≈ 1.6 s) were observed, indicating the lack of aggregation of the switch. For comparison, the properties of the molecular switch bearing no substituents and a CF_3_ group in the ortho position were as follows: peak widths at half height = 8 Hz, relaxation times ≈ 1.6 s, and Δδ = 1.8 ppm (Table S4). This compound was successfully used in ^19^F MRI phantom experiments^36^ performed in a 30/70 water/acetonitrile solution. The NMR properties of 4b in purely aqueous solution were nearly identical. This indicates that the ^19^F NMR findings for 4b have the potential to translate well to ^19^F MRI.

## Conclusions

We have successfully obtained a series of hydrazone molecular switches with the −CF_3_ group for monitoring changes in pH via ^19^F NMR. A general relationship between Hammett constants of the substituents on the p*K*_a_ of the switches was established and allowed us to design a switch suitable for the ^19^F MRI of the pH gradient in a physiologically relevant range of 5.5–7. The results can be used in the design of hydrazone-based switches of any desired p*K*_a_ for various purposes.

## Supporting Information Available

Chemicals and reagents; measurements; sample preparation; additional experimental details; materials; and methods (PDF)

## References

[ref1] Neina D. The Role of Soil pH in Plant Nutrition and Soil Remediation. Appl. Environ. Soil Sci. 2019, 2019, 1–9. 10.1155/2019/5794869.

[ref2] Luo Q.; Hossen A.; Sameen D. E.; Ahmed S.; Dai J.; Li S.; Qin W.; Liu Y. Recent Advances in the Fabrication of pH-Sensitive Indicators Films and Their Application for Food Quality Evaluation. Crit. Rev. Food Sci. Nutr. 2021, 1–17. 10.1080/10408398.2021.1959296. 34382866

[ref3] Richter A.; Paschew G.; Klatt S.; Lienig J.; Arndt K.-F.; Adler H.-J. Review on Hydrogel-Based pH Sensors and Microsensors. Sensors 2008, 8, 561–581. 10.3390/s8010561. 27879722 PMC3668326

[ref4] Briggs E. M.; Sandoval S.; Erten A.; Takeshita Y.; Kummel A. C.; Martz T. R. Solid State Sensor for Simultaneous Measurement of Total Alkalinity and pH of Seawater. ACS Sens. 2017, 2, 1302–1309. 10.1021/acssensors.7b00305. 28805369

[ref5] Kuo S.-H.; Shen C.-J.; Shen C.-F.; Cheng C.-M. Role of pH Value in Clinically Relevant Diagnosis. Diagnostics 2020, 10, 107 10.3390/diagnostics10020107. 32079129 PMC7167948

[ref6] Orouji A.; Abbasi-Moayed S.; Ghasemi F.; Hormozi-Nezhad M. R. A Wide-Range pH Indicator Based on Colorimetric Patterns of Gold@silver Nanorods. Sens. Actuators, B 2022, 358, 131479 10.1016/j.snb.2022.131479.

[ref7] Aref M.; Ranjbari E.; García-Guzmán J. J.; Hu K.; Lork A.; Crespo G. A.; Ewing A. G.; Cuartero M. Potentiometric pH Nanosensor for Intracellular Measurements: Real-Time and Continuous Assessment of Local Gradients. Anal. Chem. 2021, 93, 15744–15751. 10.1021/acs.analchem.1c03874. 34783529 PMC8637545

[ref8] Anemone A.; Consolino L.; Arena F.; Capozza M.; Longo D. L. Imaging Tumor Acidosis: A Survey of the Available Techniques for Mapping in Vivo Tumor pH. Cancer Metastasis Rev. 2019, 38, 25–49. 10.1007/s10555-019-09782-9. 30762162 PMC6647493

[ref9] Janasik D.; Krawczyk T. ^19^F MRI Probes for Multimodal Imaging. Chem. - Eur. J. 2021, 28, e202102556 10.1002/chem.202102556. 34705306

[ref10] Zhao J.; Chen J.; Ma S.; Liu Q.; Huang L.; Chen X.; Lou K.; Wang W. Recent Developments in Multimodality Fluorescence Imaging Probes. Acta Pharm. Sin. B 2018, 8, 320–338. 10.1016/j.apsb.2018.03.010. 29881672 PMC5989919

[ref11] James M. L.; Gambhir S. S. A Molecular Imaging Primer: Modalities, Imaging Agents, and Applications. Physiol. Rev. 2012, 92, 897–965. 10.1152/physrev.00049.2010. 22535898

[ref12] Pierre V. C.; Allen M. J.; Caravan P. Contrast Agents for MRI: 30+ Years and Where Are We Going?. JBIC, J. Biol. Inorg. Chem. 2014, 19, 127–131. 10.1007/s00775-013-1074-5. 24414380 PMC4075138

[ref13] Wahsner J.; Gale E. M.; Rodríguez-Rodríguez A.; Caravan P. Chemistry of MRI Contrast Agents: Current Challenges and New Frontiers. Chem. Rev. 2019, 119, 957–1057. 10.1021/acs.chemrev.8b00363. 30350585 PMC6516866

[ref14] Tirotta I.; Dichiarante V.; Pigliacelli C.; Cavallo G.; Terraneo G.; Bombelli F. B.; Metrangolo P.; Resnati G. ^19^F Magnetic Resonance Imaging (MRI): From Design of Materials to Clinical Applications. Chem. Rev. 2015, 115, 1106–1129. 10.1021/cr500286d. 25329814

[ref15] Wolters M.; Mohades S. G.; Hackeng T. M.; Post M. J.; Kooi M. E.; Backes W. H. Clinical Perspectives of Hybrid Proton-Fluorine Magnetic Resonance Imaging and Spectroscopy. Invest. Radiol. 2013, 48, 341–350. 10.1097/RLI.0b013e318277528c. 23211551

[ref16] Xie D.; Yu M.; Kadakia R. T.; Que E. L. ^19^F Magnetic Resonance Activity-Based Sensing Using Paramagnetic Metals. Acc. Chem. Res. 2020, 53, 2–10. 10.1021/acs.accounts.9b00352. 31809009

[ref17] Blahut J.; Benda L.; Kotek J.; Pintacuda G.; Hermann P. Paramagnetic Cobalt(II) Complexes with Cyclam Derivatives: Toward ^19^F MRI Contrast Agents. Inorg. Chem. 2020, 59, 10071–10082. 10.1021/acs.inorgchem.0c01216. 32633944

[ref18] Chalmers K.; DeâLuca E.; Hogg N. H. M.; Kenwright A.; Kuprov I.; Parker D.; Botta M.; Wilson J. I.; Blamire A. Design Principles and Theory of Paramagnetic Fluorine-Labelled Lanthanide Complexes as Probes for ^19^F Magnetic Resonance: A Proof-of-Concept Study. Chem. - Eur. J. 2010, 16, 134–148. 10.1002/chem.200902300. 19957317

[ref19] Yu J.-X. X.; Hallac R. R.; Chiguru S.; Mason R. P. New Frontiers and Developing Applications in ^19^F NMR. Prog. Nucl. Magn. Reson. Spectrosc. 2013, 70, 25–49. 10.1016/j.pnmrs.2012.10.001. 23540575 PMC3613763

[ref20] Carril M. Activatable Probes for Diagnosis and Biomarker Detection by MRI. J. Mater. Chem. B 2017, 5, 4332–4347. 10.1039/C7TB00093F. 32263963

[ref21] Mizukami S. Development of Molecular Imaging Tools to Investigate Protein Functions by Chemical Probe Design. Chem. Pharm. Bull. 2011, 59, 1435–1446. 10.1248/cpb.59.1435. 22130363

[ref22] Peterson K. L.; Srivastava K.; Pierre V. C. Fluorinated Paramagnetic Complexes: Sensitive and Responsive Probes for Magnetic Resonance Spectroscopy and Imaging. Front. Chem. 2018, 6, 160 10.3389/fchem.2018.00160. 29876342 PMC5974164

[ref23] Kodibagkar V. D.; Yu J.; Liu L.; Hetherington H. P.; Mason R. P. Imaging β-Galactosidase Activity Using ^19^F Chemical Shift Imaging of LacZ Gene-Reporter Molecule 2-Fluoro-4-Nitrophenol-β-d-Galactopyranoside. Magn. Reson. Imaging 2006, 24, 959–962. 10.1016/j.mri.2006.04.003. 16916713

[ref24] Harvey P.; Chalmers K. H.; De Luca E.; Mishra A.; Parker D. Paramagnetic ^19^F Chemical Shift Probes That Respond Selectively to Calcium or Citrate Levels and Signal Ester Hydrolysis. Chem. - Eur. J. 2012, 18, 8748–8757. 10.1002/chem.201200737. 22689478

[ref25] Basal L. A.; Bailey M. D.; Romero J.; Ali M. M.; Kurenbekova L.; Yustein J.; Pautler R. G.; Allen M. J. Fluorinated Eu II -Based Multimodal Contrast Agent for Temperature- and Redox-Responsive Magnetic Resonance Imaging. Chem. Sci. 2017, 8, 8345–8350. 10.1039/C7SC03142D. 29780447 PMC5933353

[ref26] Wang Z.; Su M.-Y.; Nalcioglu O. Applications of Dynamic Contrast Enhanced MRI in Oncology: Measurement of Tumor Oxygen Tension. Technol. Cancer Res. Treat. 2002, 1, 29–38. 10.1177/153303460200100104. 12614174

[ref27] Cho M. H.; Shin S. H.; Park S. H.; Kadayakkara D. K.; Kim D.; Choi Y. Targeted, Stimuli-Responsive, and Theranostic ^19^F Magnetic Resonance Imaging Probes. Bioconjugate Chem. 2019, 30, 2502–2518. 10.1021/acs.bioconjchem.9b00582. 31536323

[ref28] Tannock I. F.; Rotin D. Acid pH in Tumors and Its Potential for Therapeutic Exploitation. Cancer Res. 1989, 49, 4373–4384. 2545340

[ref29] Hao G.; Xu Z. P.; Li L. Manipulating Extracellular Tumour pH: An Effective Target for Cancer Therapy. RSC Adv. 2018, 8, 22182–22192. 10.1039/C8RA02095G. 35541713 PMC9081285

[ref30] Oishi M.; Sumitani S.; Nagasaki Y. On–Off Regulation of ^19^F Magnetic Resonance Signals Based on pH-Sensitive PEGylated Nanogels for Potential Tumor-Specific Smart ^19^F MRI Probes. Bioconjugate Chem. 2007, 18, 1379–1382. 10.1021/bc7002154. 17760418

[ref31] Yu J.-X.; Cui W.; Bourke V. A.; Mason R. P. 6-Trifluoromethylpyridoxine: Novel ^19^F NMR pH Indicator for in Vivo Detection. J. Med. Chem. 2012, 55, 6814–6821. 10.1021/jm300520q. 22775397 PMC3430128

[ref32] Chen S.; Yang Y.; Li H.; Zhou X.; Liu M. pH-Triggered Au-Fluorescent Mesoporous Silica Nanoparticles for ^19^F MR/Fluorescent Multimodal Cancer Cellular Imaging. Chem. Commun. 2014, 50, 283–285. 10.1039/C3CC47324D. 24170041

[ref33] Huang X.; Huang G.; Zhang S.; Sagiyama K.; Togao O.; Ma X.; Wang Y.; Li Y.; Soesbe T. C.; Sumer B. D.; Takahashi M.; Sherry A. D.; Gao J. Multi-Chromatic pH-Activatable ^19^F-MRI Nanoprobes with Binary ON/OFF pH Transitions and Chemical-Shift Barcodes. Angew. Chem., Int. Ed. 2013, 52, 8074–8078. 10.1002/anie.201301135. PMC3771347 23788453

[ref34] Li Y.; Zhang H.; Guo C.; Hu G.; Wang L. Multiresponsive Nanoprobes for Turn-On Fluorescence/ ^19^F MRI Dual-Modal Imaging. Anal. Chem. 2020, 92, 11739–11746. 10.1021/acs.analchem.0c01786. 32786481

[ref35] Zalewski M.; Janasik D.; Kapała A.; Minoshima M.; Sugihara F.; Raj W.; Pietrasik J.; Kikuchi K.; Krawczyk T. pH-Sensitive Polymethacrylates as Potential Contrast Agents in ^19^F MRI. Macromol. Chem. Phys. 2022, 223, 2200027 10.1002/macp.202200027.

[ref36] Janasik D.; Jasiński K.; Węglarz W. P.; Nemec I.; Jewula P.; Krawczyk T. Ratiometric pH-Responsive ^19^F Magnetic Resonance Imaging Contrast Agents Based on Hydrazone Switches. Anal. Chem. 2022, 94, 3427–3431. 10.1021/acs.analchem.1c04978. 35156816 PMC8892427

[ref37] Aprahamian I. Hydrazone Switches and Things in Between. Chem. Commun. 2017, 53, 6674–6684. 10.1039/C7CC02879B. 28540954

[ref38] Feringa B. L.; Browne W. R. Molecular Switches; WileyVCH, 2001.

[ref39] Zhang J. L.; Zhong J. Q.; Lin J. D.; Hu W. P.; Wu K.; Xu G. Q.; Wee A. T. S.; Chen W. Towards Single Molecule Switches. Chem. Soc. Rev. 2015, 44, 2998–3022. 10.1039/C4CS00377B. 25757483

[ref40] Dattler D.; Fuks G.; Heiser J.; Moulin E.; Perrot A.; Yao X.; Giuseppone N. Design of Collective Motions from Synthetic Molecular Switches, Rotors, and Motors. Chem. Rev. 2020, 120, 310–433. 10.1021/acs.chemrev.9b00288. 31869214

[ref41] Landge S. M.; Aprahamian I. A pH Activated Configurational Rotary Switch: Controlling the E/Z Isomerization in Hydrazones. J. Am. Chem. Soc. 2009, 131, 18269–18271. 10.1021/ja909149z. 19968272

[ref42] Bornhorst G. M.; Rutherfurd S. M.; Roman M. J.; Burri B. J.; Moughan P. J.; Singh R. P. Gastric pH Distribution and Mixing of Soft and Rigid Food Particles in the Stomach Using a Dual-Marker Technique. Food Biophys. 2014, 9, 292–300. 10.1007/s11483-014-9354-3.

[ref43] Su X.; Lõkov M.; Kütt A.; Leito I.; Aprahamian I. Unusual Para-Substituent Effects on the Intramolecular Hydrogen-Bond in Hydrazone-Based Switches. Chem. Commun. 2012, 48, 10490 10.1039/c2cc35860c. 22990382

[ref44] Paskaleva V.; Dobrev S.; Kochev N.; Angelova S.; Antonov L. Unusual Para-Substituent Effects on the Intramolecular Hydrogen Bond in Hydrazone-Based Switches: Insights from Chemical Landscape Analysis and DFT Calculations. Physchem 2021, 1, 189–201. 10.3390/physchem1020013.

[ref45] Andon R. J. L.; Cox J. D.; Herington E. F. G. The Ultra-Violet Absorption Spectra and Dissociation Constants of Certain Pyridine Bases in Aqueous Solution. Trans. Faraday Soc. 1954, 50, 918–927. 10.1039/tf9545000918.

[ref46] Jiang W.; Lumata L.; Chen W.; Zhang S.; Kovacs Z.; Sherry A. D.; Khemtong C. Hyperpolarized ^15^N-Pyridine Derivatives as pH-Sensitive MRI Agents. Sci. Rep. 2015, 5, 9104 10.1038/srep09104. 25774436 PMC4360734

[ref47] Hansch C.; Leo A.; Taft R. W. A Survey of Hammett Substituent Constants and Resonance and Field Parameters. Chem. Rev. 1991, 91, 165–195. 10.1021/cr00002a004.

