# Supplementary material for: Tuning the pH of Activation of Fluorinated Hydrazone-Based Switches—A Pathway to Versatile 19F Magnetic Resonance Imaging Contrast Agents
Source: ACS Sens. 2023 Jan 25;8(2):721–7. doi: 10.1021/acssensors.2c02251 (PMC9972467; doi:10.1021/acssensors.2c02251)
Supplement: Supplementary file 1 — se2c02251_si_001.pdf [file se2c02251_si_001.pdf]

Supporting information

Tuning the pH of Activation of Fluorinated Hydrazone-Based Switches – a pathway to versatile ^{19}F magnetic resonance imaging contrast agents.

Dawid Janasik, Patrycja Imielska, Tomasz Krawczyk*

Department of Chemical Organic Technology and Petrochemistry, Silesian University of Technology Krzywoustego 4, 44-100 Gliwice, Poland

Synthesis

Pirydyl acetates p-H, p-Cl and p-CH_3

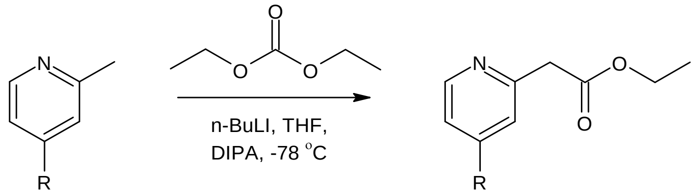

Ethyl 2-(pyridin-2-yl)acetate derivatives were synthesized following the procedure: n-BuLi (2.5 M solution in hexanes, 2.05 eq.) was added dropwise to a stirred solution of diisopropylamine (2.10 eq.) in THF at -78°C under Ar. The resulting solution was warmed to 0°C and stirred at 0°C for 1 h. Then, the solution was transferred to a stirred solution of modified 2-picoline (1.0 eq) and diethyl carbonate (3.0 eq.) in THF at -78°C under Ar. The resulting solution was stirred at -78°C for 1 h and then allowed to warm to room temperature and stirred for 30 min. Saturated $NH_4Cl_{(aq.)}$ and water were added, and the two layers were separated, and the aqueous layer was extracted with Et_2O. The combined organic layers were dried with $MgSO_4$ and evaporated under reduced pressure to give the product (Yeld 70-85%) as a bright yellow oil.

p-H: ^{1}H NMR (400 MHz, $CDCl_3$, 298 K): δ 8.54 (d, J = 7.0 Hz 1H), 7.64 (t, J = 7.0 Hz, 1H), 7.28 (d, J = 7.0 Hz, 1H), 7.17 (dd, J = 7.0, 5.0 Hz, 1H), 4.17 (q, J = 7.0 Hz, 2H), 3.82 (s, 2H), 1.24 (t, J = 7.0 Hz, 3H); ^{13}C NMR (100 MHz, $CDCl_3$) δ 170.7, 154.5, 149.5, 136.7, 123.9, 122.1, 61.1, 40.0, 14.2. HR-MS (ESI): m/z calcd. For $C_9H_{11}NO_2$, $[M–H]^+$, 166,0789; found 166,1891. Yield = 85%.

p-Cl: HR-MS (ESI): m/z calcd. for $C_9H_{10}NO_2Cl$, $[M–H]^+$, 200,0478; found 200,0466. Yield = 75%.

p-CH_3: HR-MS (ESI): m/z calcd. for $C_{10}H_{13}NO_2$, $[M–H]^+$, 180,1024; found 180,1023. Yield = 80%.

Hydrazones 1a-1g, 2a, 2b, 3a, 3b, 4a

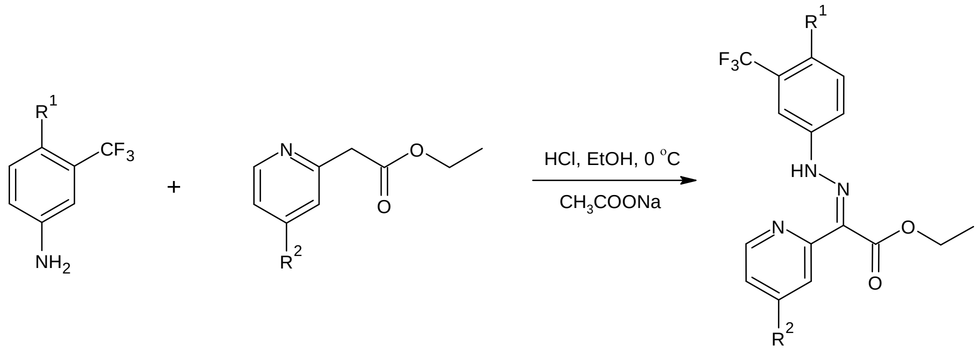

Trifluoromethylaniline 1 eq was dissolved in a mixture of conc. HCl and EtOH and stirred in an ice bath for 30 min. A cold solution of sodium nitrite (1 eq) was then added dropwise over a period of 30 min. The obtained solution of diazonium salt was then added dropwise to a suspension of ethyl-2-pyridylacetate (1 eq) and sodium acetate (6.4 eq) in a cooled (0°C) ethanol/water (8:1) mixture. The resultant reaction mixture was stirred overnight and then washed with methylene chloride. The organic fraction was washed twice with saturated sodium bicarbonate solution and dried over magnesium sulfate. The crude product was then subjected to silica gel column chromatography (hexane with ethyl acetate) to give the pure compounds (Yeld 30-70%).

1a (ethyl (2E)-{2-[4-nitro-3-(trifluoromethyl)phenyl]hydrazinylidene}(pyridin-2-yl)acetate): ^{1}H NMR (400 MHz, $CDCl_3$, 298 K): δ = 15.18 (s, 1H, NH), 8.70 (ddd, J = 4.8, 1.6, 0.8 Hz, 1H), 8.18 (dt, J = 8.4, 1.0 Hz, 1H), 8.09 (dd, J = 8.8, 3.2 Hz, 1H), 7.90 (ddd, J = 9.6, 7.0, 1.5 Hz, 1H), 7.70 (d, J = 2.4 Hz, 1H), 7.55 (dd, J = 8.8, 2.4 Hz, 1H), 7.40 (ddd, J = 7.6, 4.8, 1.0 Hz, 1H), 4.43 (q, J = 7.2 Hz, 2H), 1.46 (t, J = 7.2 Hz, 3H); ^{13}C NMR (100 MHz, $CDCl_3$): δ 162.63, 149.28, 145.03, 144.54, 138.85, 135.17, 128.41, 125.83, 125.13, 122.84, 121.40, 118.37, 114.04, 111.22 (q, J = 6.1 Hz), 59.42, 20.38; HR-MS (ESI): m/z calcd. for $C_{16}H_{13}N_4O_4F_3$, $[M-H]^+$, 383.0967; found 383.0958. Yield = 46%.

1b (ethyl (2E)-{2-[4-cyano-3-(trifluoromethyl)phenyl]hydrazinylidene}(pyridin-2-yl)acetate): ^{1}H NMR (400 MHz, $CDCl_3$, 298 K): δ = 15.12 (s, 1H, NH), 8.69 (ddd, J = 4.8, 2.0, 0.9 Hz, 1H), 8.17 (dt, J = 8.4, 1.0 Hz, 1H), 7.90 (ddd, J = 9.6, 7.1, 1.2 Hz, 1H), 7.75 (d, J = 8.4 Hz, 1H), 7.68 (d, J = 2.0 Hz, 1H), 7.55 (dd, J = 8.4, 2.2 Hz, 1H), 7.39 (ddd, J = 7.2, 5.0, 1.1 Hz, 1H), 4.44 (q, J = 7.1 Hz, 2H), 1.46 (t, J = 7.2 Hz, 3H); ^{13}C NMR (100 MHz, $CDCl_3$): δ 162.40, 149.17, 144.35, 134.98, 133.66, 127.91, 125.75, 122.65, 121.17, 120.93, 119.86, 114.29, 113.80, 110.17 (q, J = 4.8 Hz), 99.04, 59.23, 11.79; HR-MS (ESI): m/z calcd. for $C_{17}H_{13}N_4O_2F_3$, $[M-H]^+$, 363.1069; found 363.1066. Yield = 66%.

1c (ethyl (2E)-{2-[4-bromo-3-(trifluoromethyl)phenyl]hydrazinylidene}(pyridin-2-yl)acetate): ^{1}H NMR (400 MHz, $CDCl_3$, 298 K): δ = 14.92 (s, 1H, NH), 8.66 (ddd, J = 5.2, 1.8, 0.9 Hz, 1H), 8.21 (dt, J = 8.0, 1.0 Hz, 1H), 7.85 (ddd, J = 9.2, 7.0, 1.3

Hz, 1H), 7.65 (d, *J* = 2.8 Hz, 1H), 7.62 (d, *J* = 8.4 Hz, 1H), 7.39 (dd, *J* = 8.4, 2.6 Hz, 1H), 7.32 (ddd, *J* = 7.6, 5.2, 1.0 Hz, 1H), 4.40 (q, *J* = 7.1 Hz, 2H), 1.45 (t, *J* = 7.0 Hz, 3H); ^{13}C NMR (100 MHz, $CDCl_3$): δ 165.26, 152.25, 146.57, 142.82, 137.11, 135.65, 130.67, 127.57, 124.67, 124.19, 122.92, 121.47, 118.53, 114.20 (q, *J* = 5.7 Hz), 61.34, 14.29; HR-MS (ESI): m/z calcd. for $C_{16}H_{13}N_3O_2F_3Br$, [M-H]$^+$, 416.0222; found 416.0226. Yield = 58%.

1d (ethyl (2E)-{2-[4-chloro-3-(trifluoromethyl)phenyl]hydrazinylidene}(pyridin-2-yl)acetate): ^{1}H NMR (400 MHz, $CDCl_3$, 298 K): δ = 14.93 (s, 1H, NH), 8.66 (ddd, *J* = 4.8, 1.8, 0.8 Hz, 1H), 8.23 (dt, *J* = 8.4, 1.0 Hz, 1H), 7.84 (ddd, *J* = 9.6, 7.6, 1.4 Hz, 1H), 7.64 (d, *J* = 2.4 Hz, 1H), 7.52 (m, *J* = 14.2, 2.5 Hz, 1H), 7.45 (d, *J* = 2.0 Hz, 1H), 7.32 (ddd, *J* = 7.6, 4.8, 1.2 Hz, 1H), 7.25 (s, 1H), 4.40 (q, *J* = 6.8 Hz, 2H), 1.45 (t, *J* = 7.2 Hz, 3H); ^{13}C NMR (100 MHz, $CDCl_3$): δ 165.28, 152.27, 146.56, 142.27, 137.10, 132.46, 132.25, 127.46, 124.65, 124.14, 122.90, 121.42, 118.37, 113.82 (q, *J* = 5.6 Hz), 61.34, 14.30; HR-MS (ESI): m/z calcd. for $C_{16}H_{13}N_3O_2F_3Cl$, [M-H]$^+$, 372.0727; found 372.0731. Yield = 67%.

1e (ethyl (2E)-{2-[3-(trifluoromethyl)phenyl]hydrazinylidene}(pyridin-2-yl)acetate): ^{1}H NMR (400 MHz, $CDCl_3$, 298 K): δ = 14.89 (s, 1H, NH), 8.66 (ddd, *J* = 5.0, 2.0, 0.5 Hz, 1H), 8.23 (dt, *J* = 8.0, 2.0 Hz, 1H), 7.83 (td, *J* = 7.5, 1.5 Hz, 1H), 7.58 (s, 1H), 7.54 (d, *J* = 8.0 Hz, 1H), 7.44 (t, *J* = 8.0 Hz, 1H), 7.30 (ddd, *J* = 7.9, 2.1, 0.5 Hz, 1H), 7.25 (d, *J* = 7.9 Hz, 1H), 4.40 (q, *J* = 7.1 Hz, 2H), 1.45 (t, *J* =7.1 Hz, 3H); ^{13}C NMR (100 MHz, $CDCl_3$) δ 165.4, 152.4, 146.5, 143.9, 136.9, 131.5 (q, *J* = 3.1 Hz), 129.8, 126.9, 124.5, 123.4, 122.7, 118.9, 117.7, 111.5, 61.2, 14.3; HR-MS (ESI): m/z calcd. for $C_{16}H_{14}N_3O_2F_3$, [M-H]$^+$, 338.1116; found 338.1119. Yield = 63%.

1f (ethyl (2E)-{2-[4-methylo-3-(trifluoromethyl)phenyl]hydrazinylidene}(pyridin-2-yl)acetate): ^{1}H NMR (400 MHz, $CDCl_3$, 298 K): δ = 14.78 (s, 1H, NH), 8.58 (ddd, *J* = 4.8, 2.0, 0.9 Hz, 1H), 8.18 (dt, *J* = 8.4, 0.9 Hz, 1H), 7.75 (ddd, *J* = 9.6, 7.0, 1.4 Hz, 1H), 7.51 (d, *J* = 2.4 Hz, 1H), 7.36 (dd, *J* = 8.0, 2.2 Hz, 1H), 7.22 (ddd, *J* = 7.6, 4.8, 1.1 Hz, 1H), 7.18 (d, *J* = 8.4 Hz, 1H), 4.34 (q, *J* = 7.2 Hz, 2H), 2.37 (d, *J* =1.6 Hz, 3H), 1.38 (t, *J* = 7.0 Hz, 3H); ^{13}C NMR (100 MHz, $CDCl_3$): δ 165.53, 152.61, 146.46, 141.46, 136.90, 132.87, 130.24, 129.80, 126.12, 125.79, 124.42, 122.48, 117.45, 112.45 (q, *J* = 6.0 Hz), 61.14, 18.68, 14.32; HR-MS (ESI): m/z calcd. for $C_{17}H_{16}N_3O_2F_3$, [M-H]$^+$, 352.1273; found 352.1283. Yield = 36%.

1g (ethyl (2E)-{2-[4-methoxy-3-(trifluoromethyl)phenyl]hydrazinylidene}(pyridin-2-yl)acetate): ^{1}H NMR (400 MHz, $CDCl_3$, 298 K): δ = 14.91 (s, 1H, NH), 8.64 (ddd, *J* = 4.8, 1.8, 1.0 Hz, 1H), 8.25 (dt, *J* = 8.4, 1.0 Hz, 1H), 7.82 (ddd, *J* = 9.6, 7.0, 1.4 Hz, 1H), 7.56 (d, *J* = 2.4 Hz, 1H), 7.52 (dd, *J* = 8.8, 2.8 Hz, 1H), 7.27 (ddd, *J* = 7.6, 4.8, 1.2 Hz, 1H), 7.03 (d, *J* = 9.2 Hz, 1H), 4.40 (q, *J* = 7.2 Hz, 2H), 3.90 (s, 3H), 1.44 (t, *J* = 7.0 Hz, 3H); ^{13}C NMR (100 MHz, $CDCl_3$): δ 165.59, 153.22, 152.75, 148.69, 146.34, 136.86, 136.77, 125.43, 124.84, 124.28, 122.32, 118.94, 113.98 (q, *J* = 5.5 Hz), 113.39, 61.06, 56.43, 14.34; HR-MS (ESI): m/z calcd. for $C_{17}H_{16}N_3O_3F_3$, [M-H]$^+$, 368.1222; found 368.1219. Yield = 66%.

2a (ethyl (2E)-(4-chloropyridin-2-yl){2-[3-(trifluoromethyl)phenyl]hydrazinylidene}acetate): ^{1}H NMR (400 MHz, $CDCl_3$, 298 K): δ = 14.79 (s, 1H, NH), 8.54 (dd, *J* = 4.8, 0.8 Hz, 1H), 8.39 (dd, *J* = 1.2, 0.8 Hz, 1H), 7.59 (s, 1H), 7.55 (d, *J* = 8.4 Hz, 1H), 7.46 (t, *J* = 8.0 Hz, 1H), 7.32 (dd, *J* = 3.2, 2.0 Hz, 1H), 7.29 (d, *J* = 7.6 Hz, 1H), 4.40 (q, *J* = 7.1 Hz, 2H), 1.46 (t, *J* = 7.2 Hz, 3H); ^{13}C NMR (100 MHz, $CDCl_3$): δ 165.15, 153.46, 149.62, 147.33, 145.21, 143.57, 131.64, 129.88, 125.27, 124.66, 123.01, 119.52, 117.96, 111.77 (q, *J* = 3.9 Hz), 61.36, 14.31; HR-MS (ESI): m/z calcd. for $C_{16}H_{13}N_3O_2F_3Cl$, [M-H]$^+$, 372.0727; found 372.0731. Yield = 62%.

2b (ethyl (2E)-(4-methylopyridin-2-yl){2-[3-(trifluoromethyl)phenyl]hydrazinylidene}acetate): ^{1}H NMR (400 MHz, $CDCl_3$, 298 K): δ = 14.95 (s, 1H, NH), 8.51 (d, *J* = 5.2 Hz, 1H), 8.04 (s, 1H), 7.57 (s, 1H), 7.51 (d, *J* = 8.0 Hz, 1H), 7.43 (t, *J* = 8.0 Hz, 1H), 7.25 (d, *J* = 7.6 Hz, 1H), 7.13 (d, *J* = 6.0 Hz, 1H), 4.42 (q, *J* = 7.2 Hz, 2H), 2.43 (s, 3H), 1.46 (t, *J* = 7.2 Hz, 3H); ^{13}C NMR (100 MHz, $CDCl_3$): δ 164.53, 151.14, 147.30, 145.22, 142.92, 130.81, 130.49, 128.73, 126.08, 124.43, 122.77, 117.81, 116.65, 110.41 (q, *J* = 4.0 Hz), 60.17, 20.32, 13.30; HR-MS (ESI): m/z calcd. for $C_{17}H_{16}N_3O_2F_3$, [M-H]$^+$, 352.1273; found 352.1279. Yield = 57%.

3a (ethyl (2E)-(4-methylpyridin-2-yl){2-[4-methyl-3-(trifluoromethyl)phenyl]hydrazinylidene}acetate): ^{1}H NMR (400 MHz, $CDCl_3$, 298 K): δ = 14.91 (s, 1H, NH), 8.51 (d, *J* = 5.2 Hz, 1H), 8.06 (dd, *J* = 1.6, 0.8 Hz, 1H), 7.57 (d, *J* = 2.4 Hz, 1H), 7.43 (dd, *J* = 8.4, 2.4 Hz, 1H), 7.24 (d, *J* = 8.4 Hz, 1H), 7.12 (ddd, *J* = 5.2, 1.2, 0.6 Hz, 1H), 4.39 (q, *J* = 7.1 Hz, 2H), 2.44 (d, J = 1.6 Hz,3H), 2.42 (s, 3H), 1.45 (t, *J* = 7.0 Hz, 3H); ^{13}C NMR (100 MHz, $CDCl_3$): δ 160.70, 153.75, 147.41, 143.26, 141.24, 136.59, 127.89, 125.10, 124.82, 121.34, 119.98, 118.65, 112.44, 107.44 (q, *J* = 4.8 Hz), 56.15, 24.76, 16.58, 13.73; HR-MS (ESI): m/z calcd. for $C_{18}H_{18}N_3O_2F_3$, [M-H]$^+$, 366.1429; found 366.1436. Yield = 36%.

3b (ethyl (2E)-(4-methylpyridin-2-yl){2-[4-methoxy-3-(trifluoromethyl)phenyl]hydrazinylidene}acetate): ^{1}H NMR (400 MHz, $CDCl_3$, 298 K): δ = 14.97 (s, 1H, NH), 8.48 (dt, *J* = 5.2, 0.4 Hz, 1H), 8.08 (dt, *J* = 10.8, 0.8 Hz, 1H), 7.56 (d, *J* = 2.8 Hz, 1H), 7.50 (dd, *J* = 8.8, 2.6 Hz, 1H), 7.11 (ddd, *J* = 5.2, 1.6, 0.8 Hz, 1H), 7.02 (d, *J* = 8.8 Hz, 1H), 4.38 (q, *J* = 7.1 Hz, 2H), 3.90 (s, 3H), 2.42 (s, 3H), 1.45 (t, *J* = 7.0 Hz, 3H); ^{13}C NMR (100 MHz, $CDCl_3$): δ 165.69, 152.51, 148.23, 148.15, 146.07, 141.40, 136.88, 125.59, 124.78, 123.45, 122.15, 118.87, 113.96 (q, *J* = 3.8 Hz), 113.39, 61.02, 56.44, 21.55, 14.35; HR-MS (ESI): m/z calcd. for $C_{18}H_{18}N_3O_3F_3$, [M-H]$^+$, 382.1378; found 382.1380. Yield = 42%.

4a (ethyl (2E)-(4-methylpyridin-2-yl){2-[4-nitro-2-(trifluoromethyl)phenyl]hydrazinylidene}acetate): ^{1}H NMR (400 MHz, $CDCl_3$, 298 K): δ = 8.34 (m, 1H), 7.96 (m, 2H), 7.71 (t, *J* = 2.8 Hz, 1H), 7.21 (m, 1H), 7.09 (m, 1H), 4.43 (q, *J* = 7.1 Hz, 2H), 3.77 (s, 1H), 2.43 (s, 3H), 1.44 (t, *J* = 7.0 Hz, 3H); ^{13}C NMR (100 MHz, $CDCl_3$): δ = 164.7, 150.5, 149.1, 146.2, 131.0, 130.3,

128.5, 128.2, 126.6, 125.4, 125.0, 124.7, 123.2, 115.3, 61.7, 21.5, 14.3. HR-MS (ESI): m/z calcd. for $C_{17}H_{16}N_4O_4F_3$, $[M-H]^+$, 397.1124; found 397.1101.

Hydrazones 1h and 4b

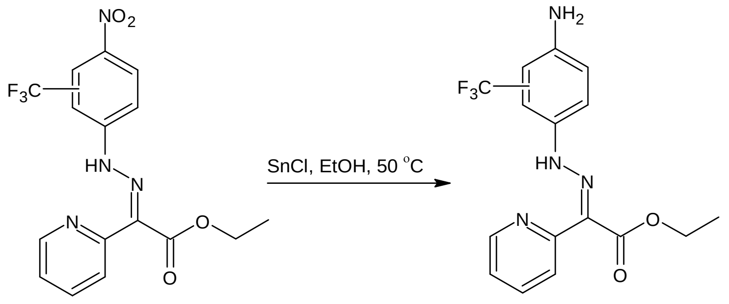

1a/4a was dissolved in ethanol and tin(II) chloride/ethanol suspension was added. The mixture was heated at 50°C for 2 h. The solvent was then removed under reduced pressure and sodium hydroxide solution was then added. Resulting mixture was steam distilled. Extraction of the distillate with chloroform gave a dark red product which was recrystallised from diethyl ether. (Yeld = 78%)

1h (ethyl (2E)-{2-[4-amino-3-(trifluoromethyl)phenyl]hydrazinylidene}(pyridin-2-yl)acetate): ^{1}H NMR (400 MHz, $CDCl_3$, 298 K): δ = 15.11 (s, 1H, NH), 8.64 (ddd, *J* = 5.2, 1.9, 0.5 Hz, 1H), 8.13 (dd, *J* = 2.3, 0.4 Hz, 1H), 8.01 (dt, *J* = 5.0, 1.9 Hz, 1H), 7.83 (d, *J* = 3 Hz, 1H), 7.64 (dd, *J* = 8.8, 2.3 Hz, 1H), 7.48 (dd, *J* = 2.3, 0.4 Hz, 1H), 7.31 (ddd, *J* = 9.6, 7.0, 1.5 Hz, 1H), 5.22 (s, 1H), 4.36 (q, *J* = 7.2 Hz, 2H), 1.39 (t, *J* = 7.1 Hz, 3H); ^{13}C NMR (100 MHz, $CDCl_3$): δ 162.95, 149.70, 145.45, 144.96, 135.59, 128.83, 126.26, 125.55, 124.52, 123.26, 121.82, 118.79, 114.47, 111.64 (q, *J* = 6.1 Hz), 59.81, 12.22; HR-MS (ESI): m/z calcd. for $C_{16}H_{15}N_4O_2F_3$, $[M-H]^+$, 353.1225; found 353.1226.

4b (ethyl (2E)-{2-[4-amino-2-(trifluoromethyl)phenyl]hydrazinylidene}(4-methylpyridin-2-yl)acetate): ^{1}H NMR (400 MHz, $(CD_3)_2SO$, 298 K): δ = 15.47 (s, 1H, NH), 8.42 (ddd, *J* = 5.2, 1.9, 0.5 Hz, 1H), 7.91 (dd, *J* = 2.3, 0.4 Hz, 1H), 7.53 (dt, *J* = 5.0, 1.9 Hz, 1H), 7.26 (d, *J* = 3 Hz, 1H), 7.16 (m, 2H), 4.27 (q, *J* = 7.2 Hz, 2H), 2.36 (s, 3H), 1.29 (t, *J* = 7.1 Hz, 3H); ^{13}C NMR (100 MHz, $(CD_3)_2SO$): δ = 169.7, 156.4, 154.5, 153.7, 150.9, 134.9, 130.8 (q, *J* = 6.2 Hz), 129.1, 128.5, 124.4, 122.4, 116.5, 115.3, 114.4, 66.5, 26.1, 19.3. HR-MS (ESI): m/z calcd. for $C_{17}H_{18}N_4O_2F_3$, $[M-H]^+$, 367.1382; found 367.1365.

NMR spectra

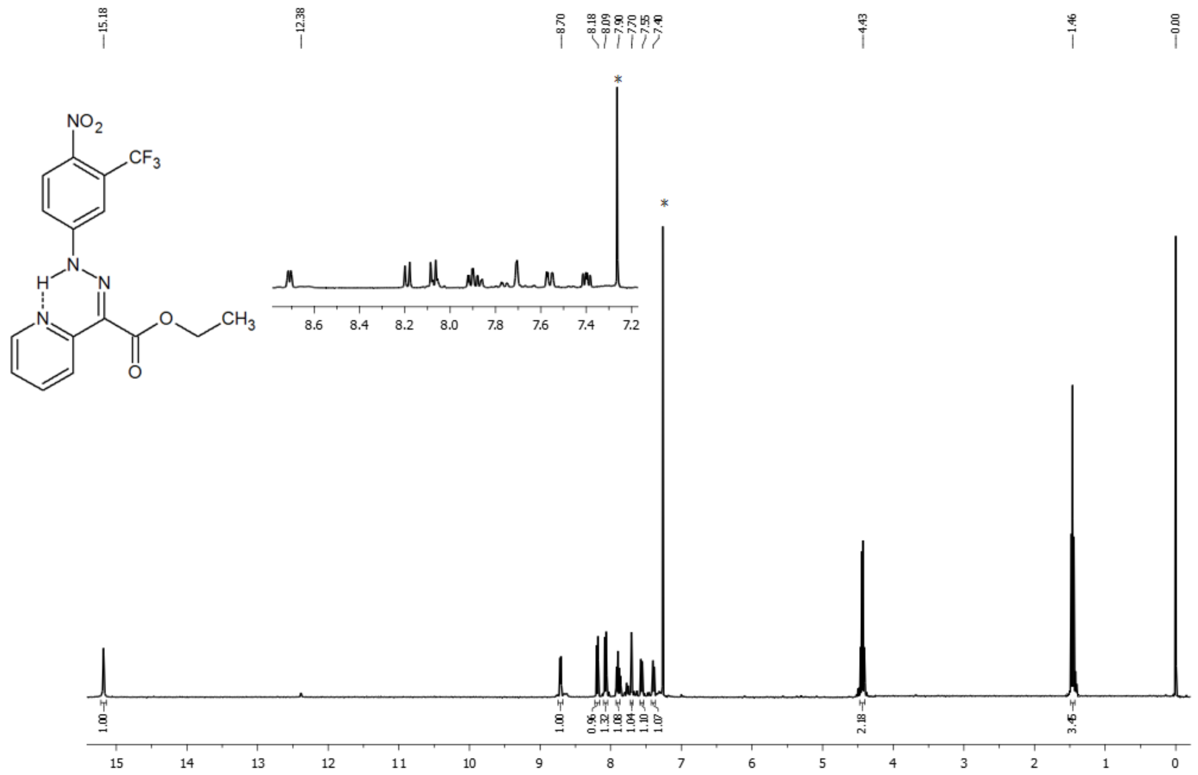

Figure S1. ^{1}H NMR spectrum of 1a in $CDCl_3$ at 298 K.

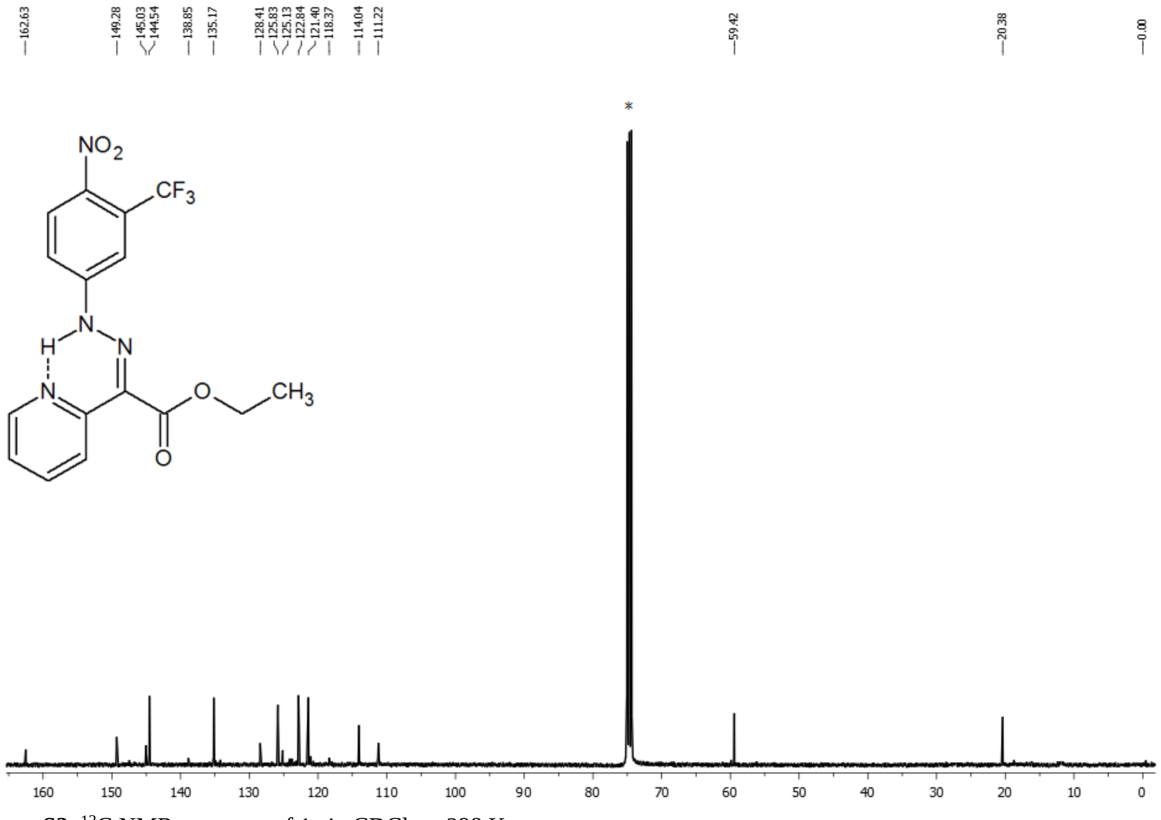

Figure S2. ^{13}C NMR spectrum of 1a in $CDCl_3$ at 298 K.

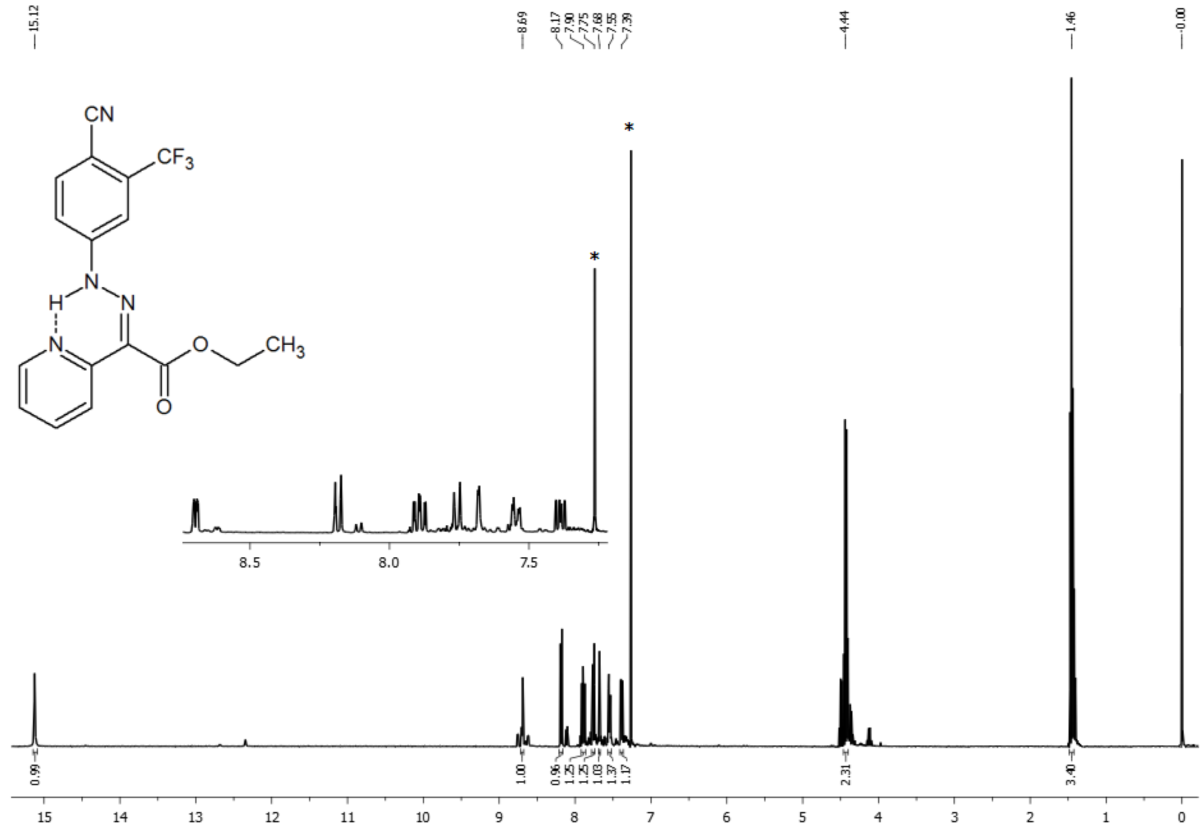

Figure S3. ^{1}H NMR spectrum of 1b in $CDCl_3$ at 298 K.

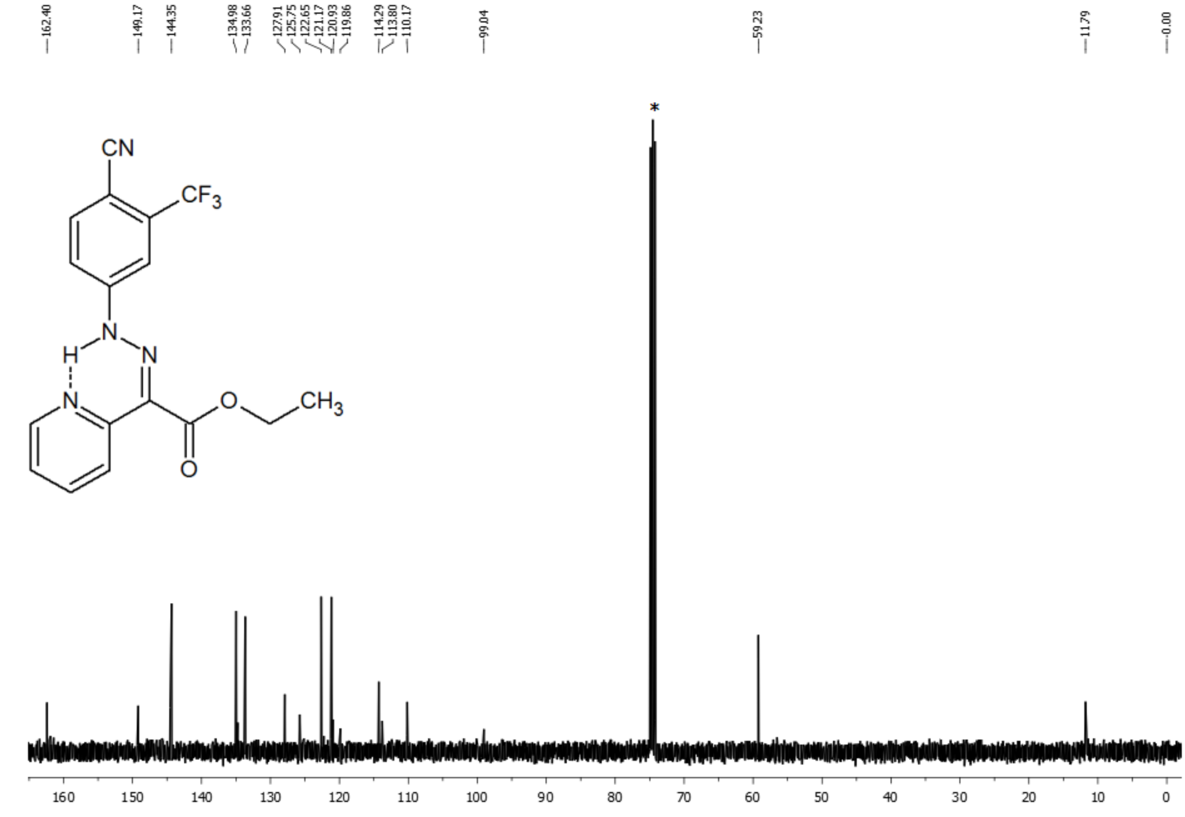

Figure S4. ^{13}C NMR spectrum of 1b in $CDCl_3$ at 298 K.

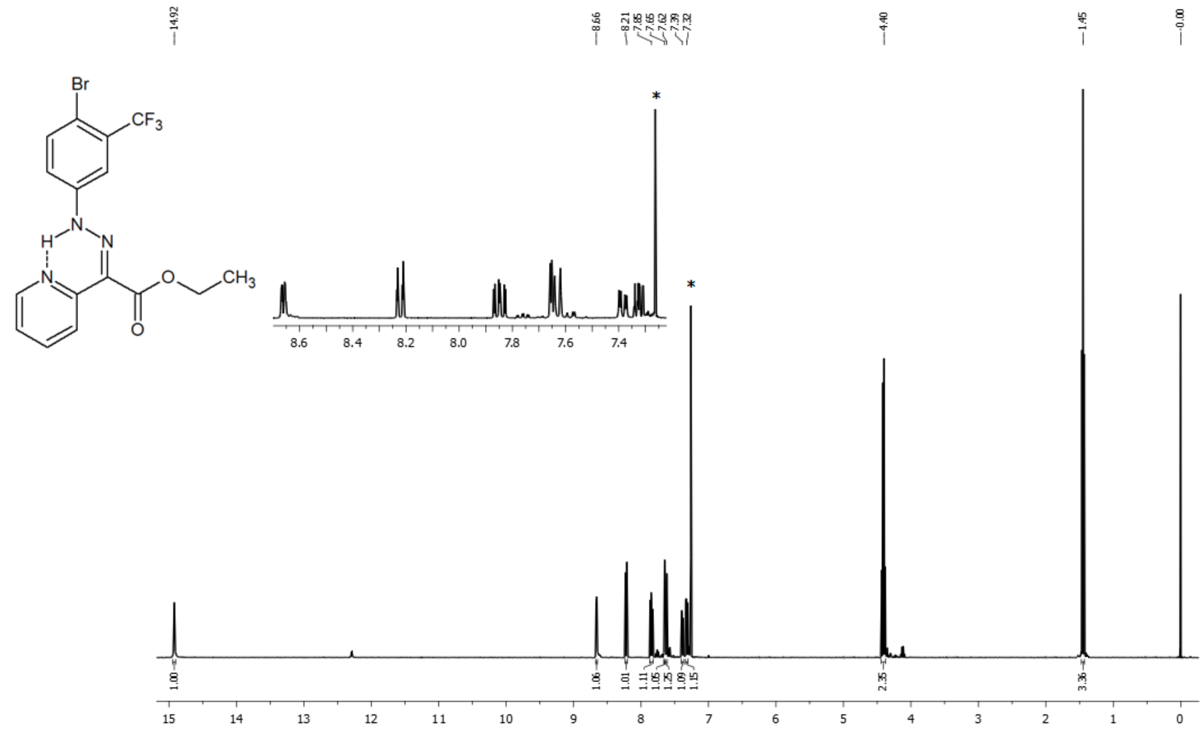

Figure S5. ^{1}H NMR spectrum of 1c in $CDCl_3$ at 298 K.

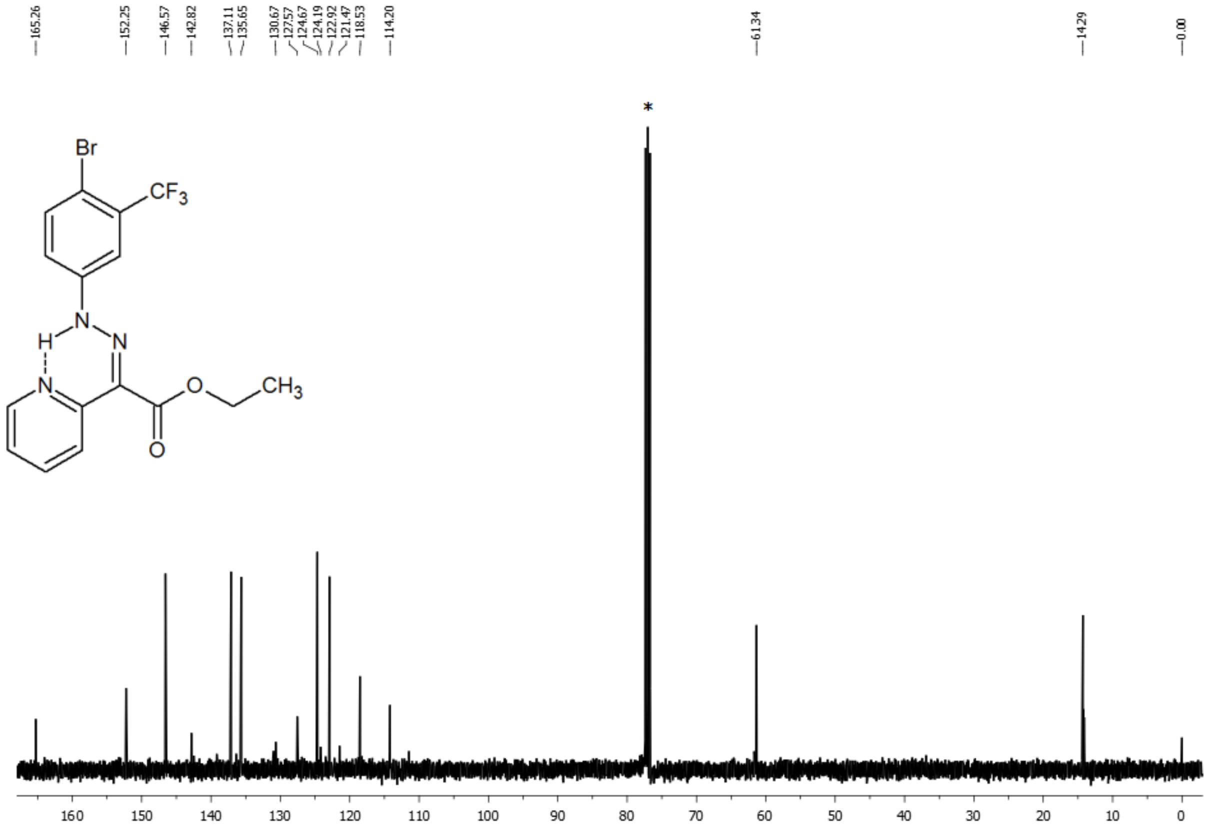

Figure S6. ^{13}C NMR spectrum of 1c in $CDCl_3$ at 298 K.

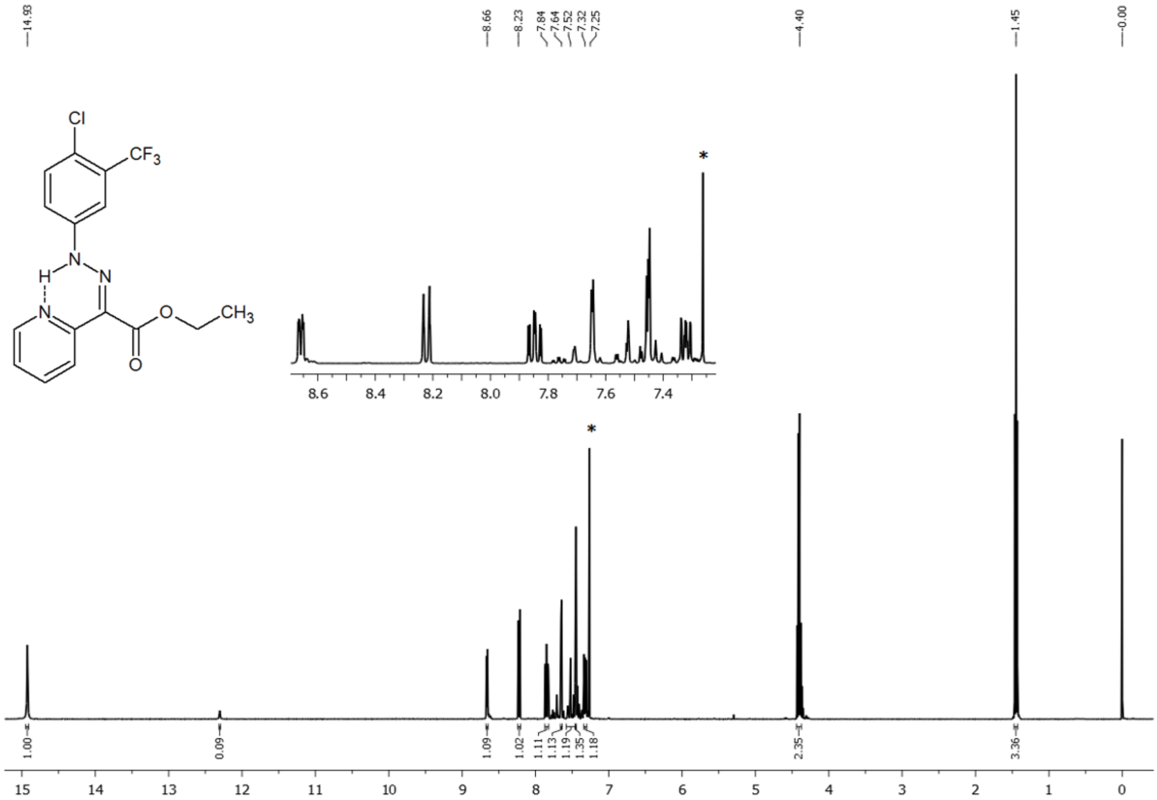

Figure S7. ^{1}H NMR spectrum of 1d in $CDCl_3$ at 298 K.

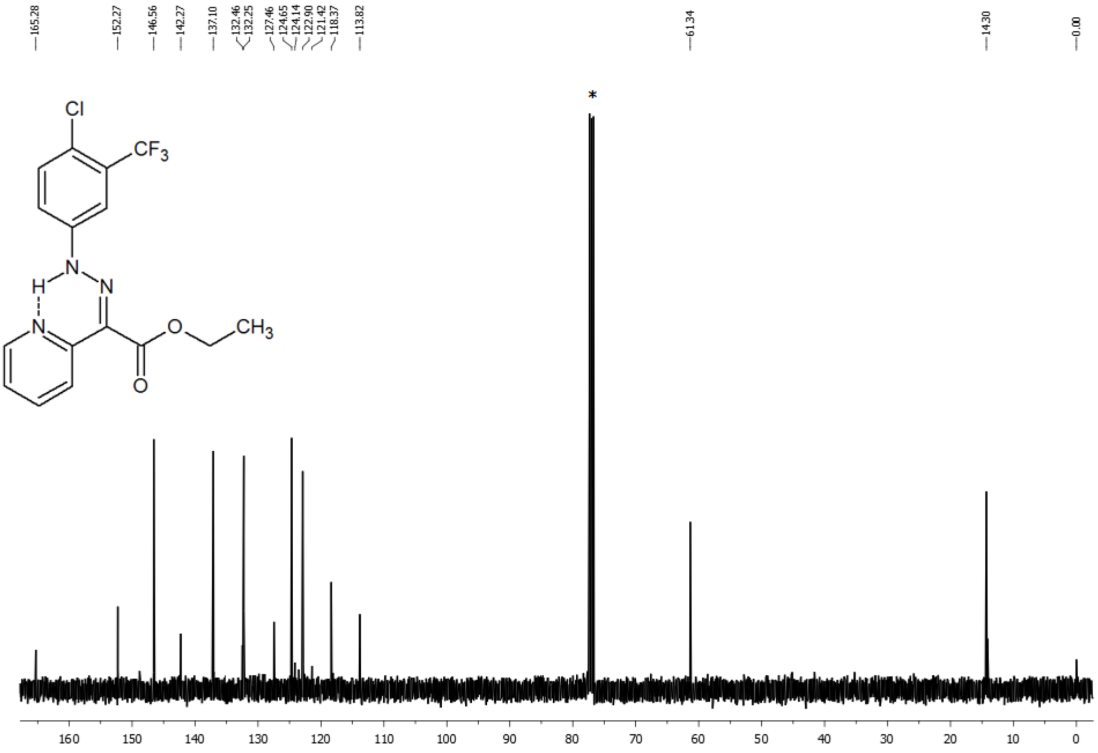

Figure S8. ^{13}C NMR spectrum of 1d in $CDCl_3$ at 298 K.

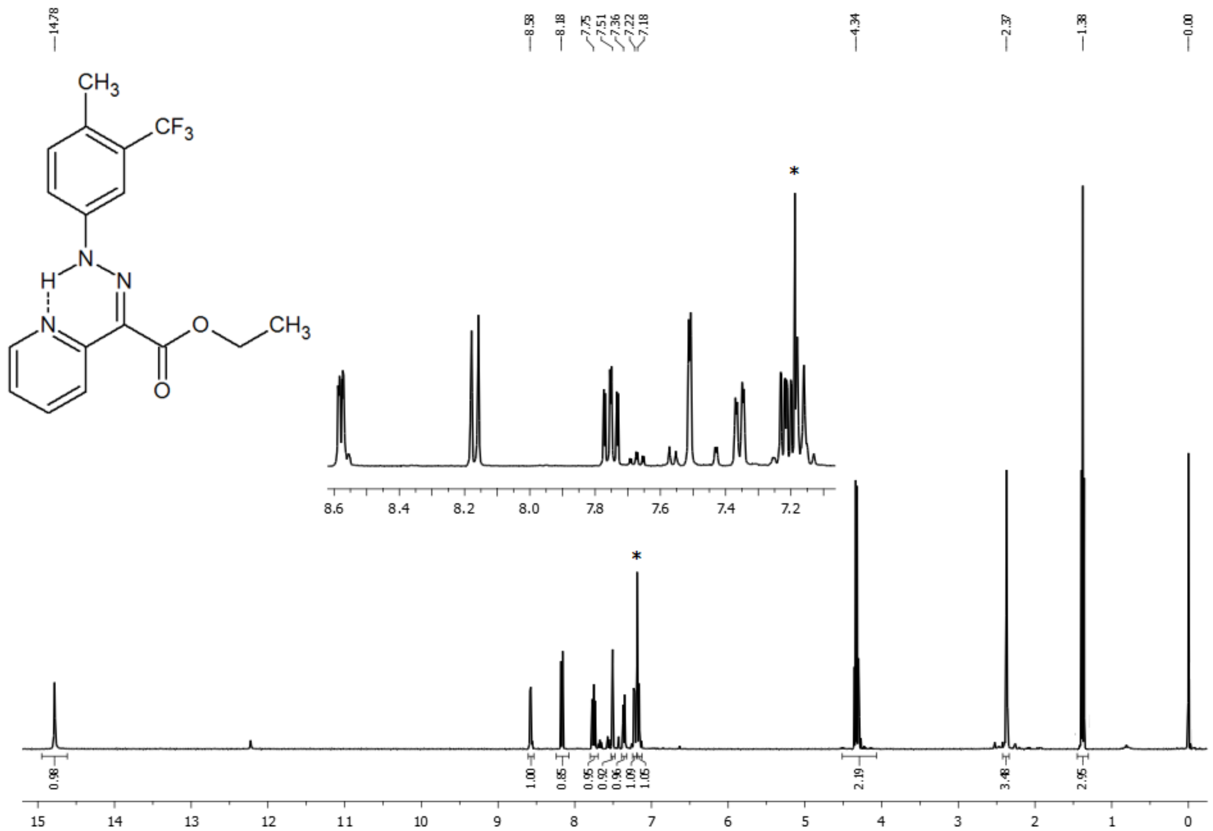

Figure S9. ^{1}H NMR spectrum of 1f in $CDCl_3$ at 298 K.

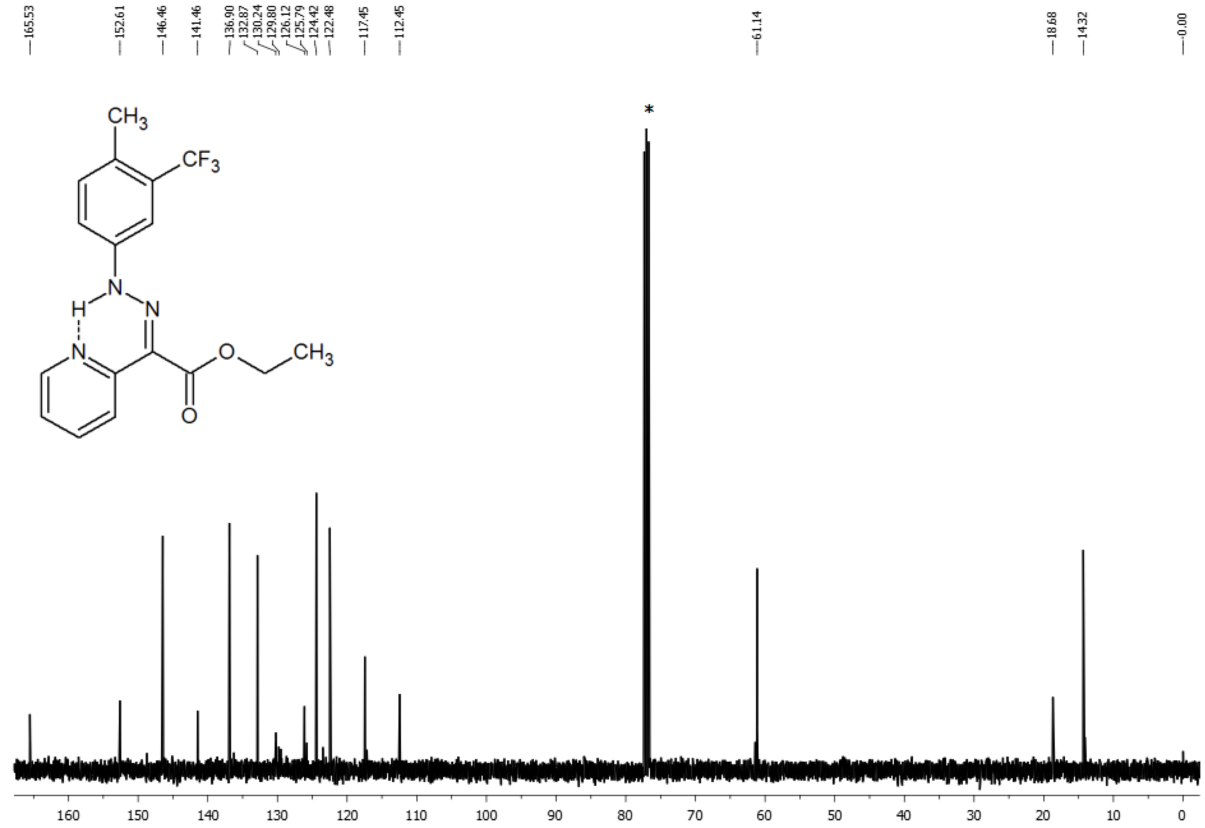

Figure S10. ^{13}C NMR spectrum of 1f in $CDCl_3$ at 298 K.

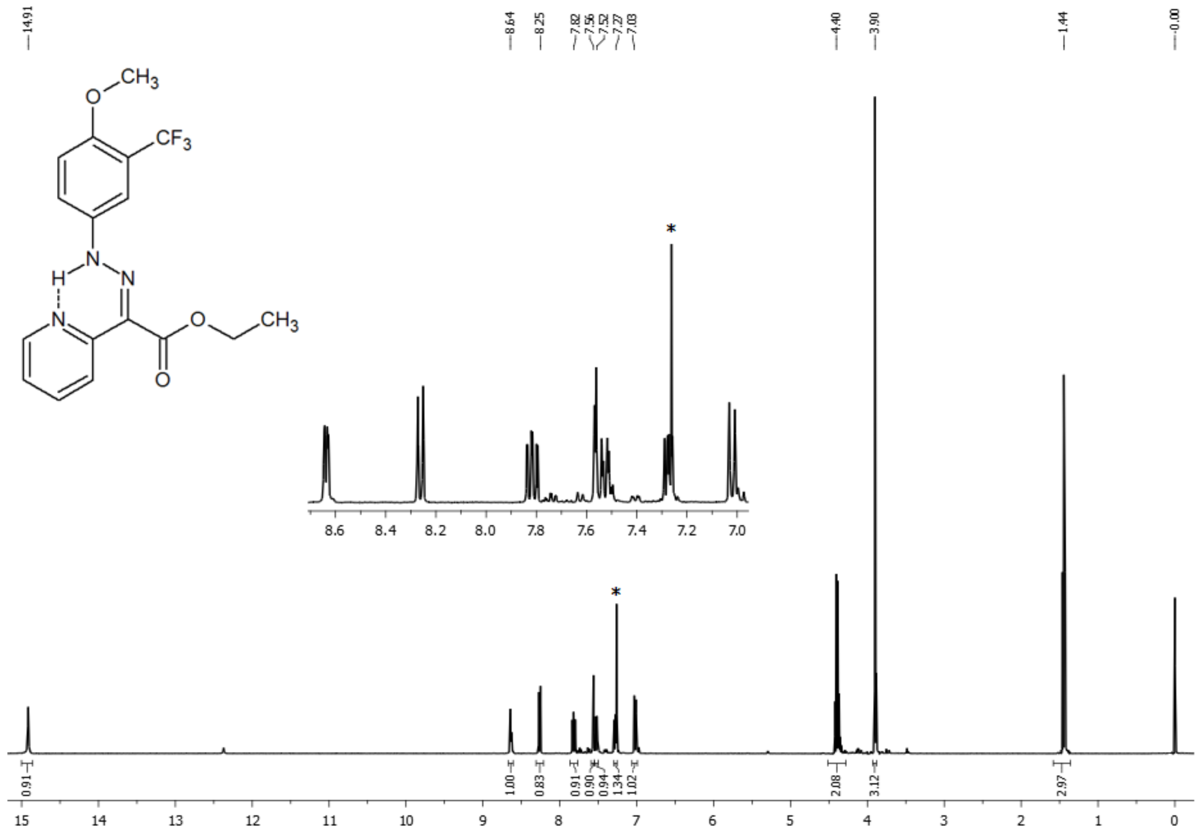

Figure S11. ^{1}H NMR spectrum of 1g in $CDCl_3$ at 298 K.

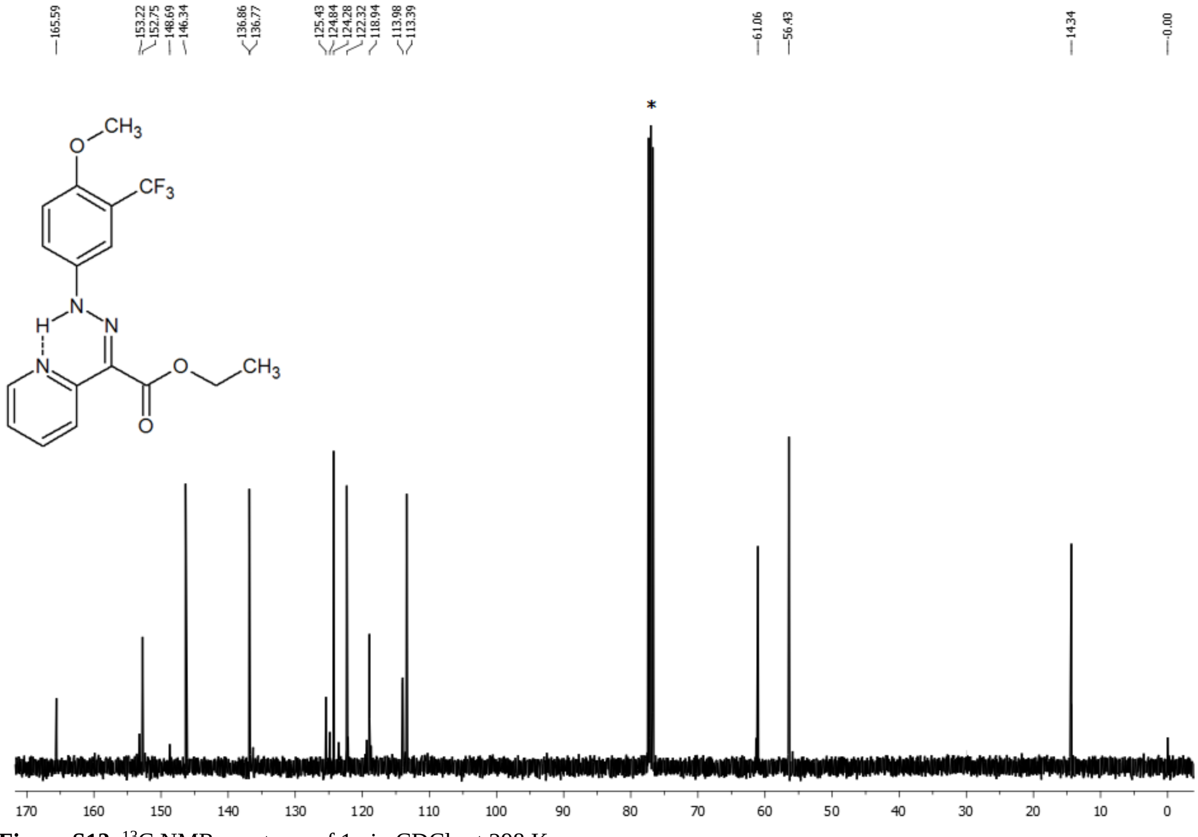

Figure S12. ^{13}C NMR spectrum of 1g in $CDCl_3$ at 298 K.

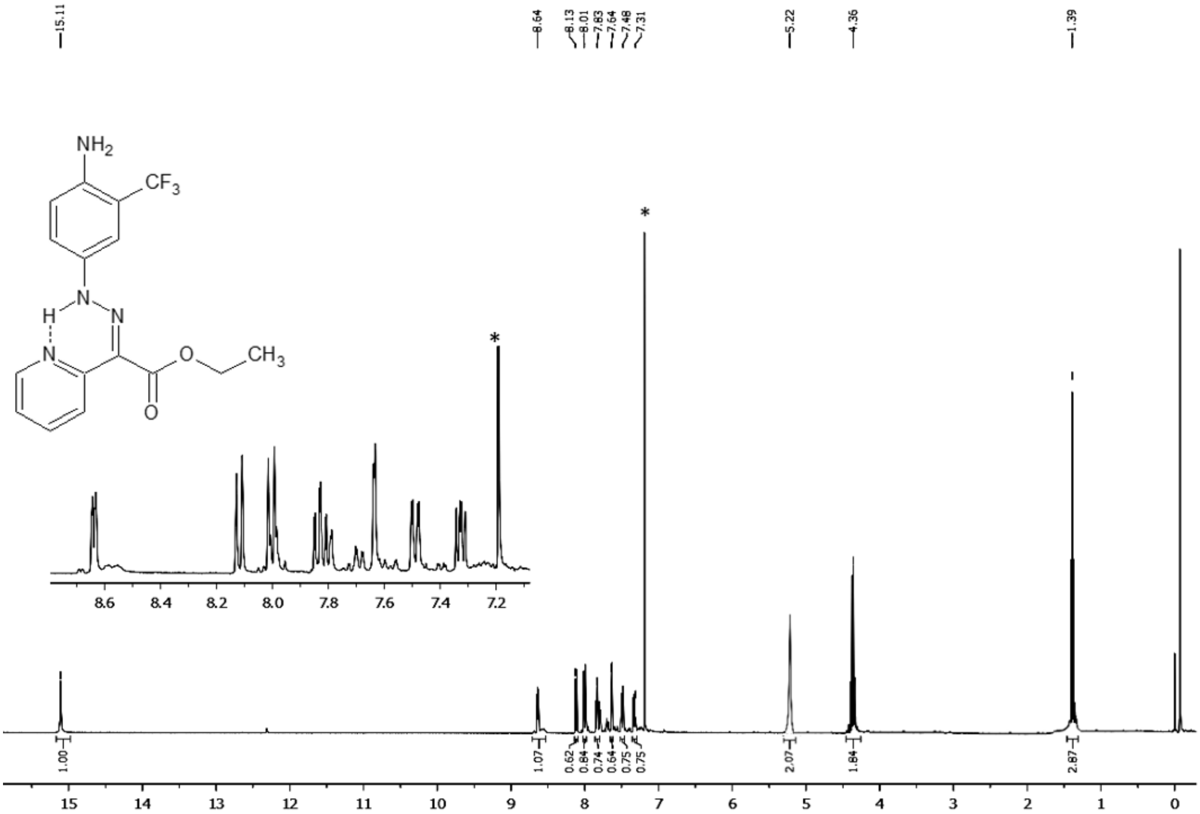

Figure S13. ^{1}H NMR spectrum of 1h in $CDCl_3$ at 298 K.

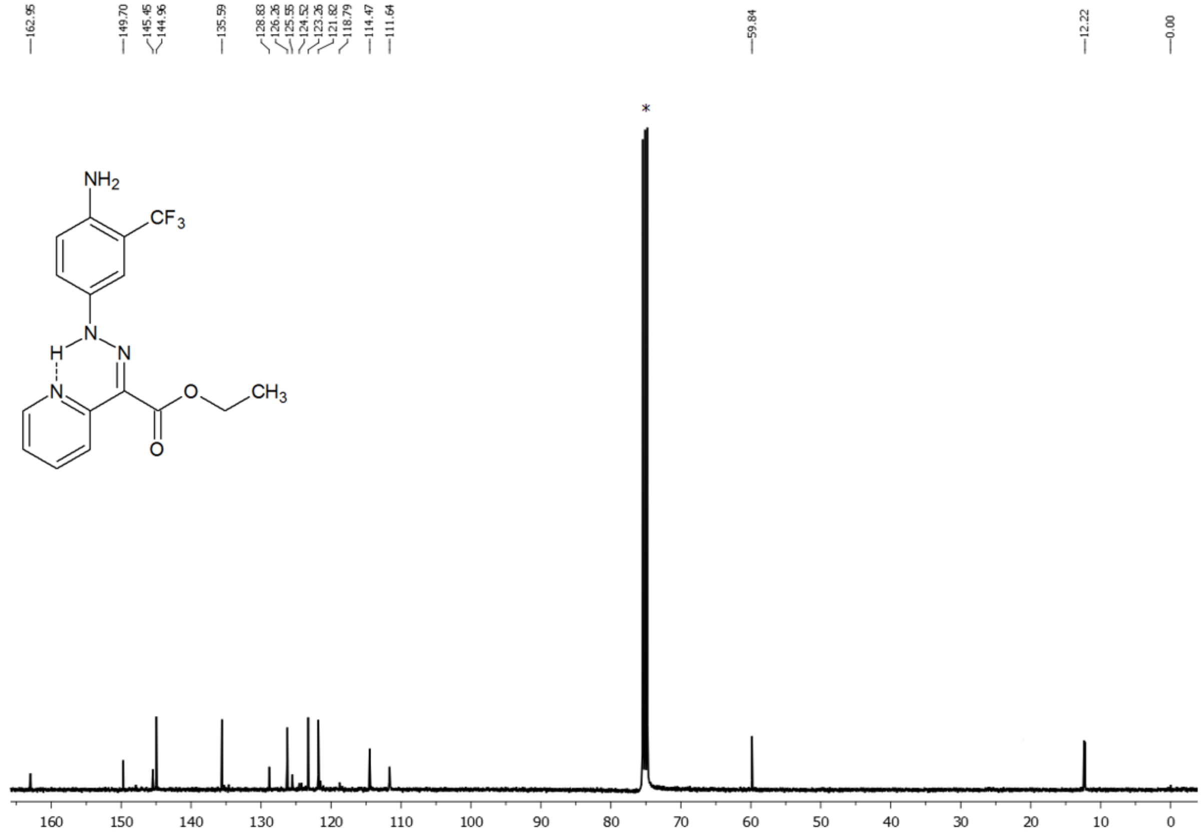

Figure S14. ^{13}C NMR spectrum of 1h in $CDCl_3$ at 298 K.

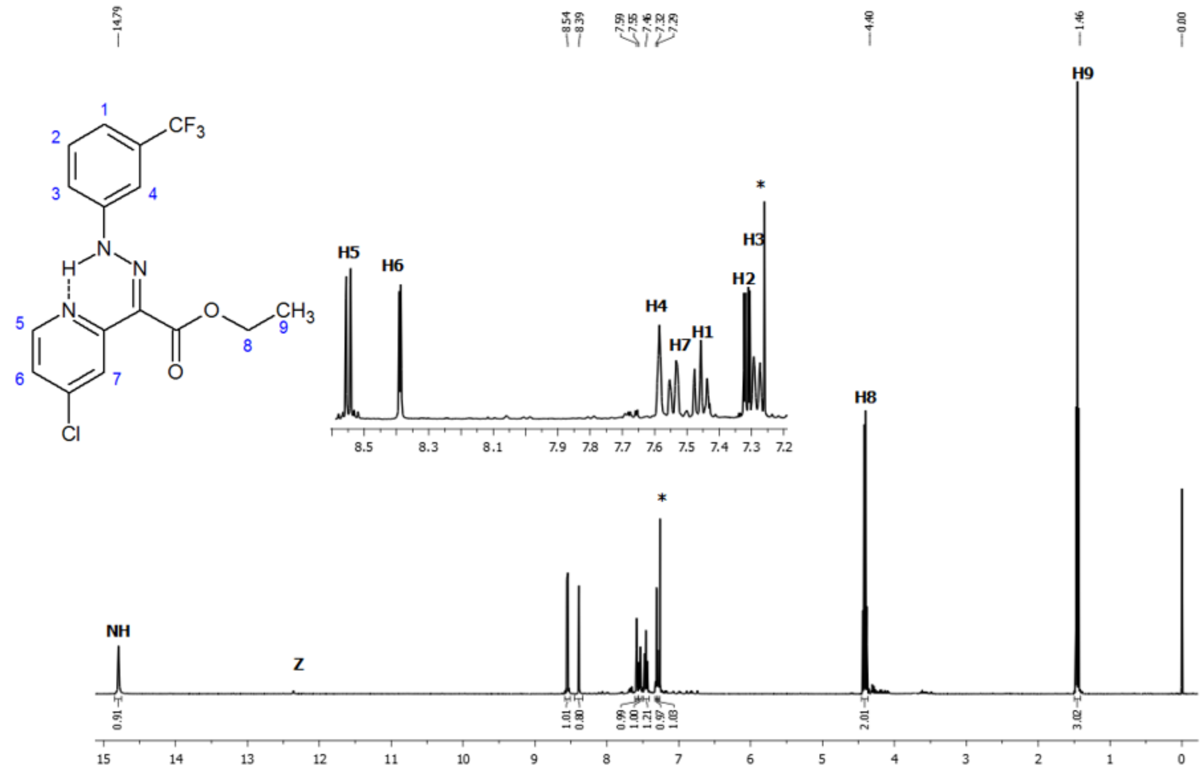

Figure S15. ^{1}H NMR spectrum of 2a in $CDCl_3$ at 298 K.

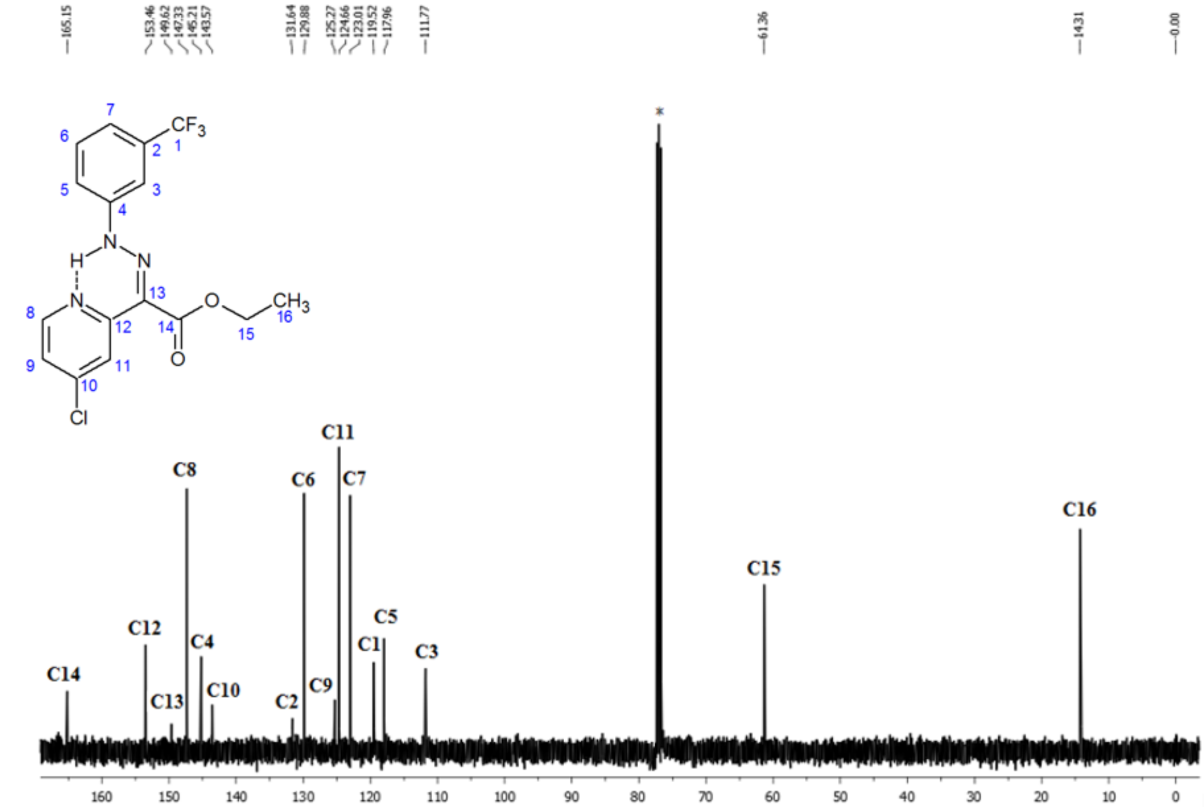

Figure S16. ^{13}C NMR spectrum of 2a in $CDCl_3$ at 298 K.

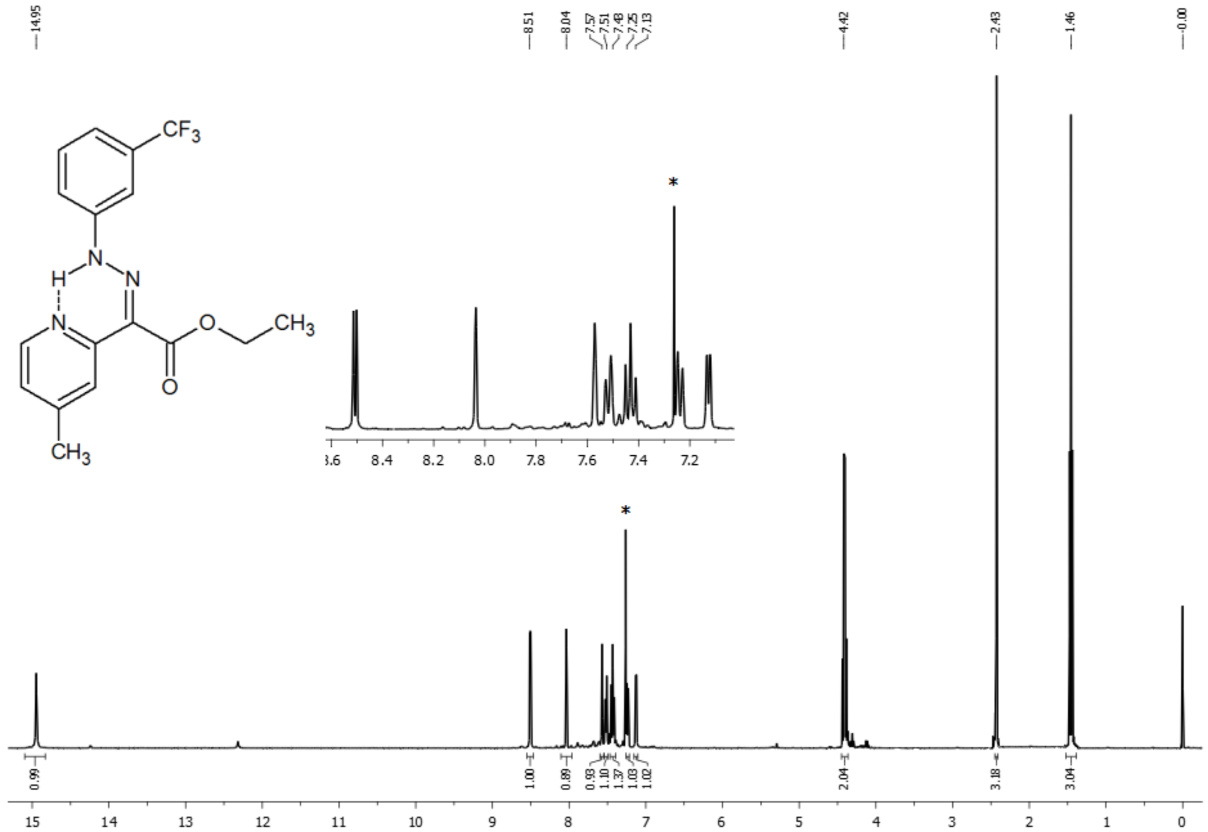

Figure S17. ^{1}H NMR spectrum of 2b in $CDCl_3$ at 298 K.

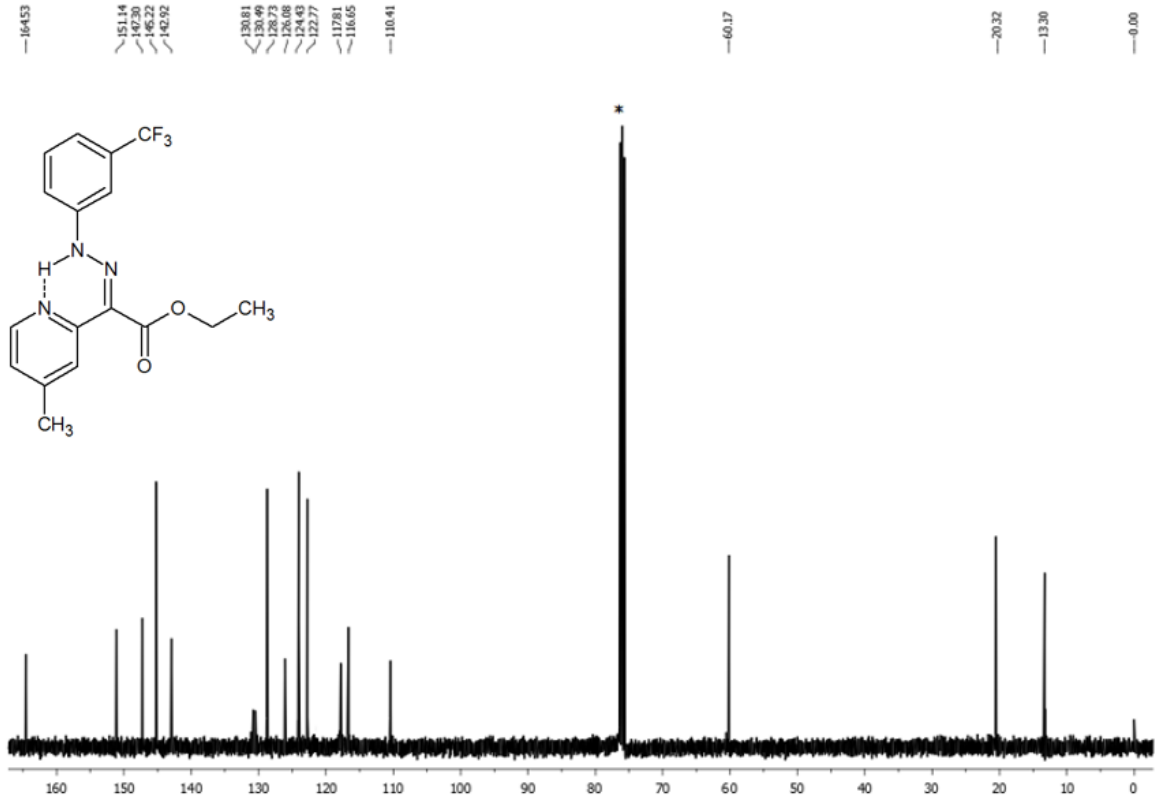

Figure S18. ^{13}C NMR spectrum of 2b in $CDCl_3$ at 298 K.

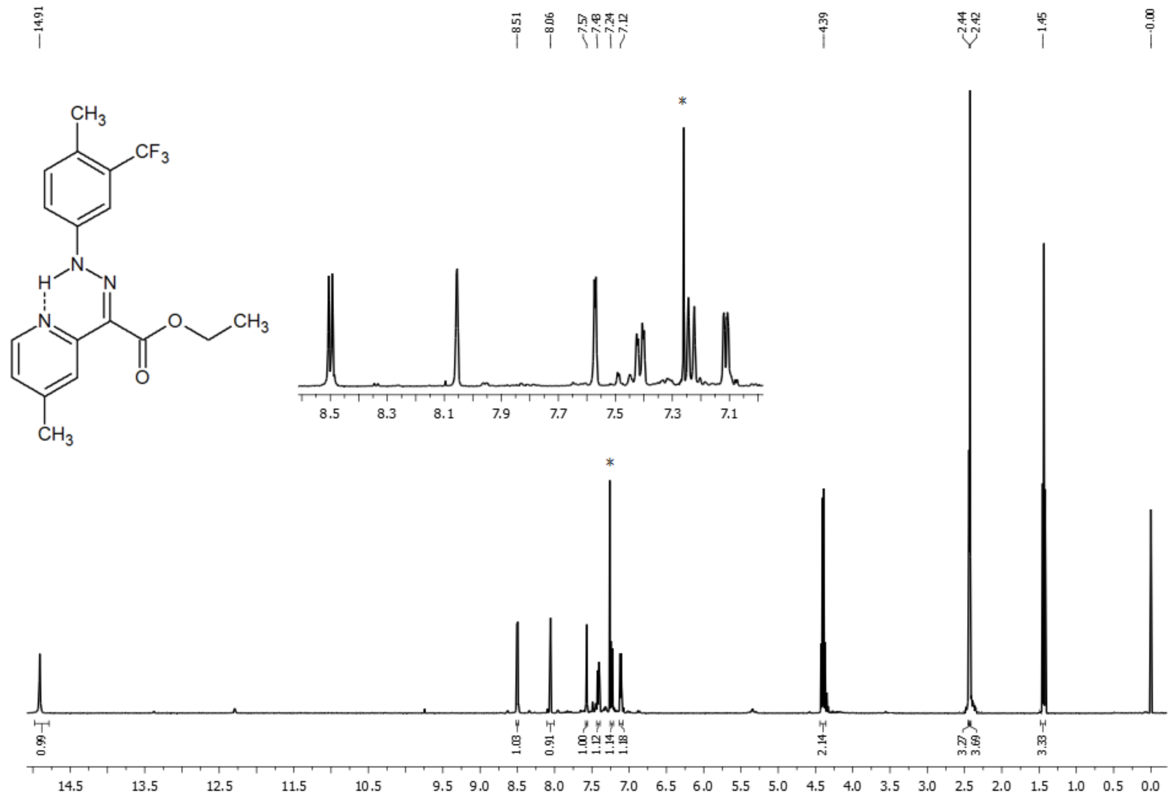

Figure S19. ^{1}H NMR spectrum of 3a in $CDCl_3$ at 298 K.

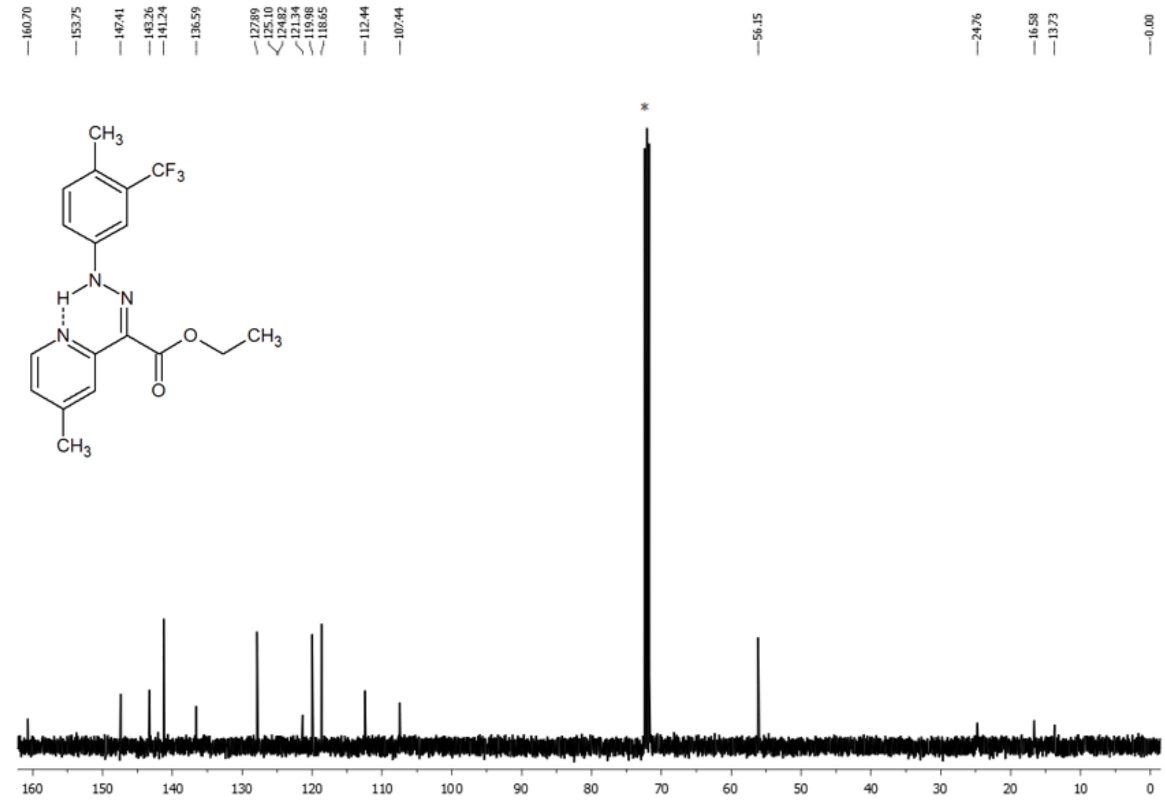

Figure S20. ^{13}C NMR spectrum of 3a in $CDCl_3$ at 298 K.

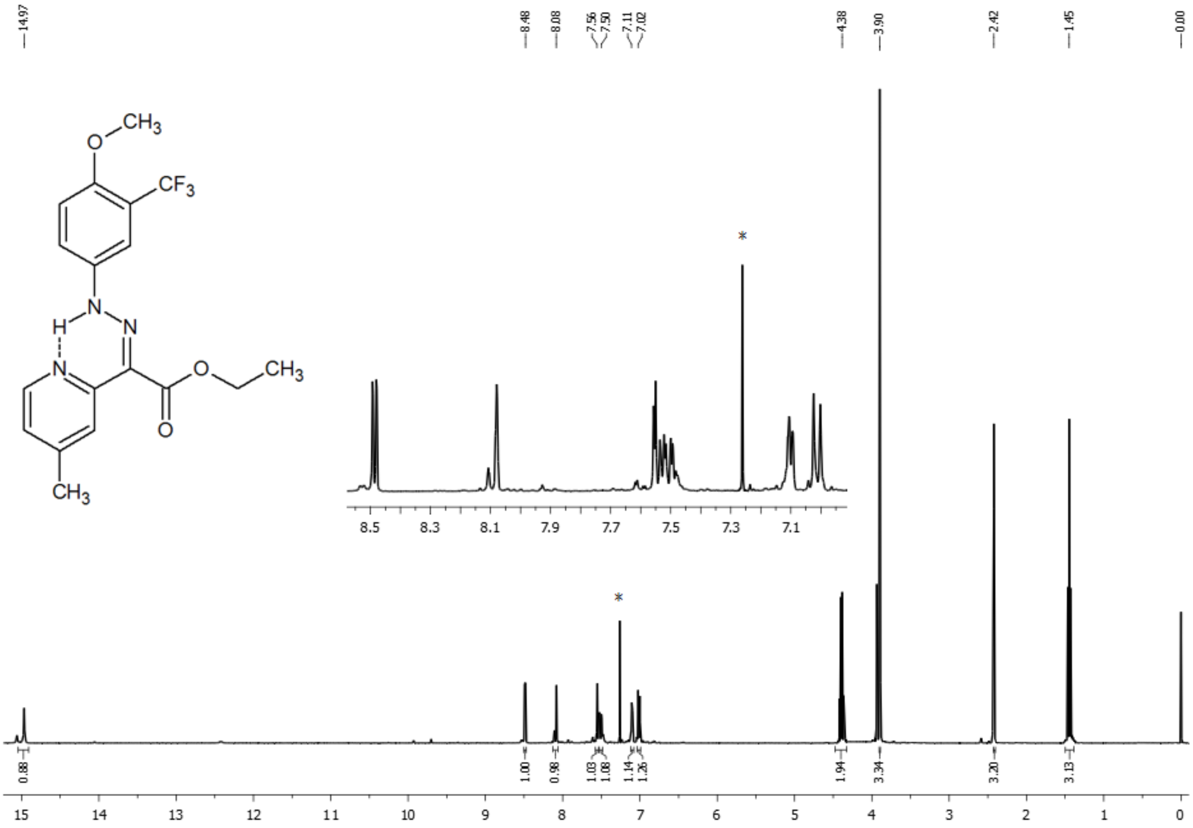

Figure S21. ^{1}H NMR spectrum of 3b in $CDCl_3$ at 298 K.

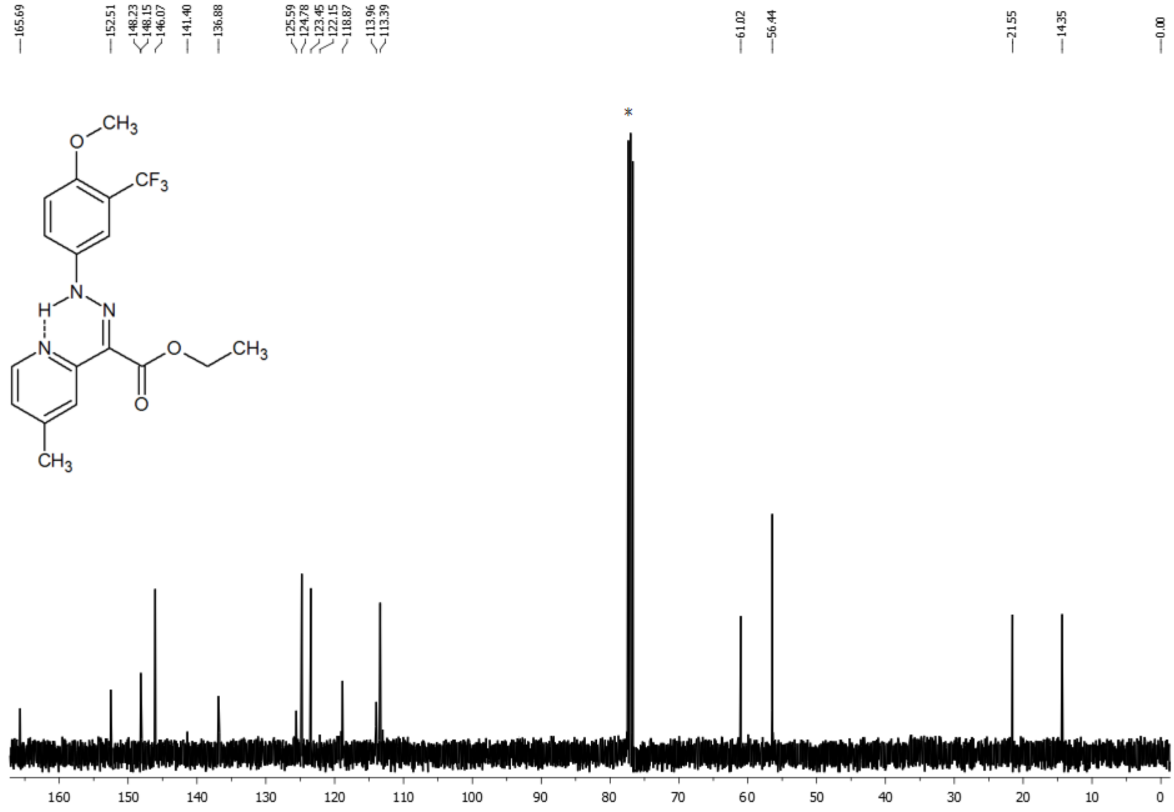

Figure S22. ^{13}C NMR spectrum of 3b in $CDCl_3$ at 298 K.

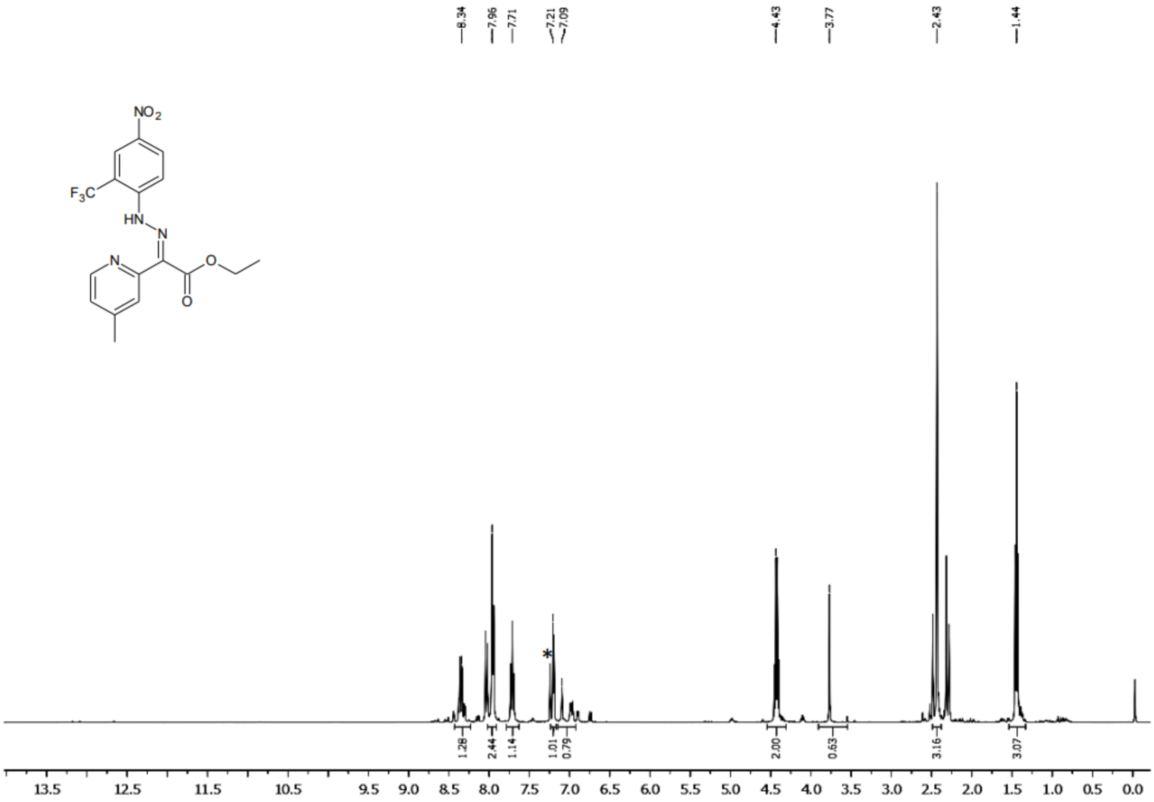

Figure S23. ^{1}H NMR spectrum of 4a in $CDCl_3$ at 298 K.

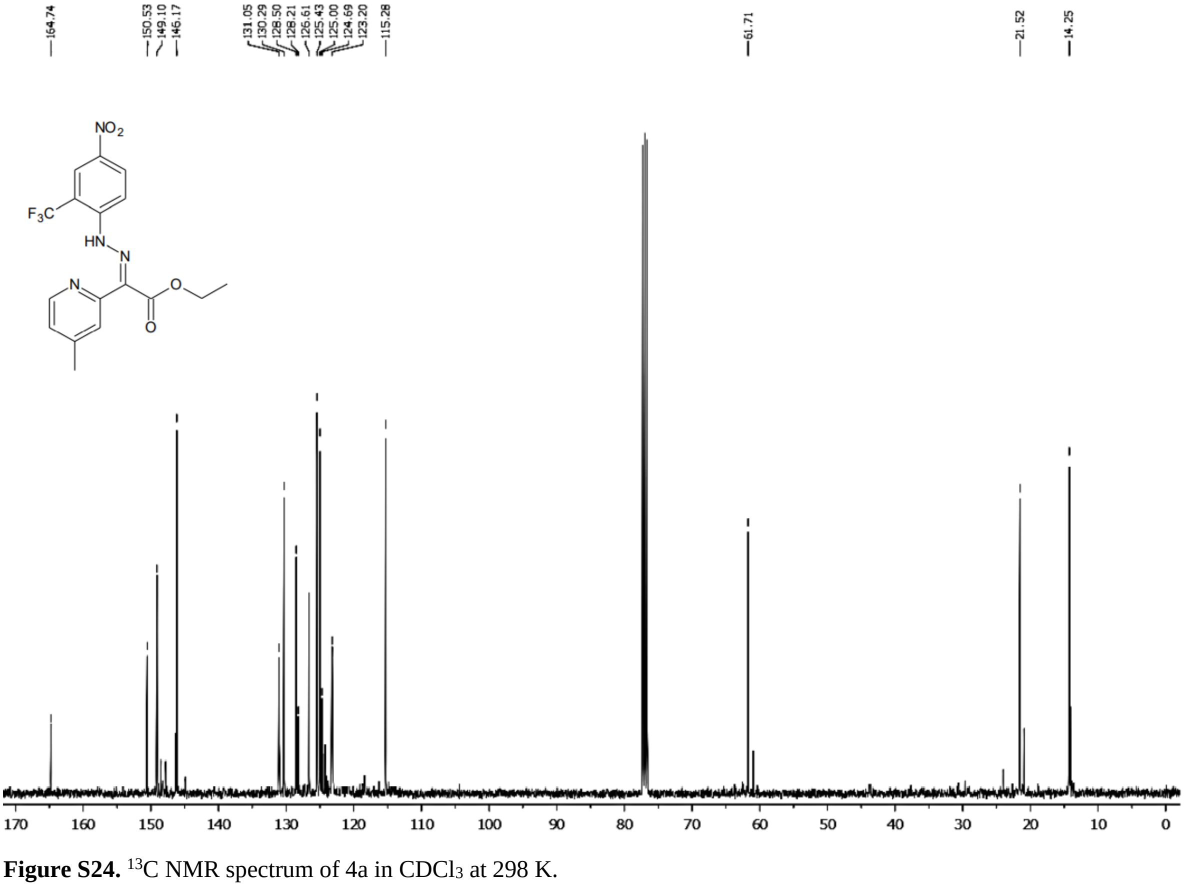

Figure S24. ^{13}C NMR spectrum of 4a in $CDCl_3$ at 298 K.

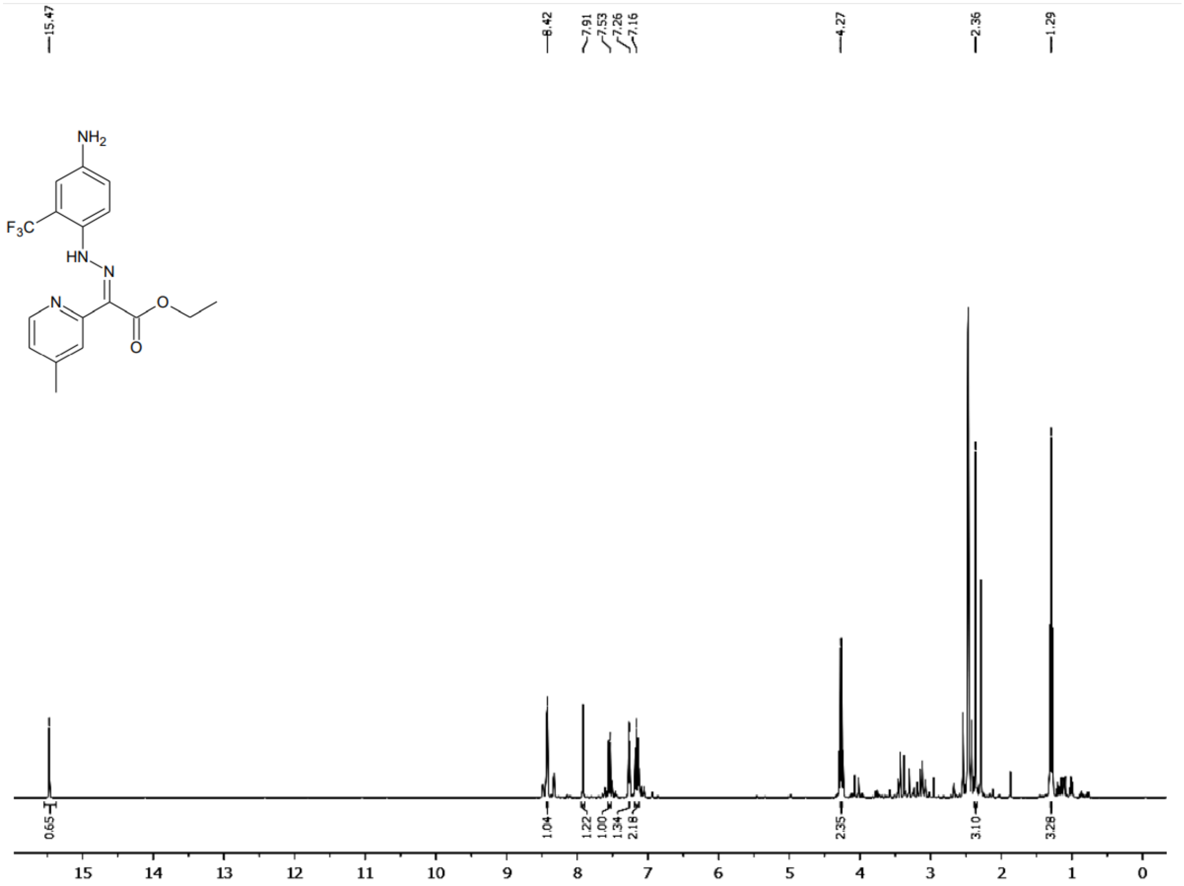

Figure S25. ^{1}H NMR spectrum of 4b in $(CD_3)_2SO$ at 298 K.

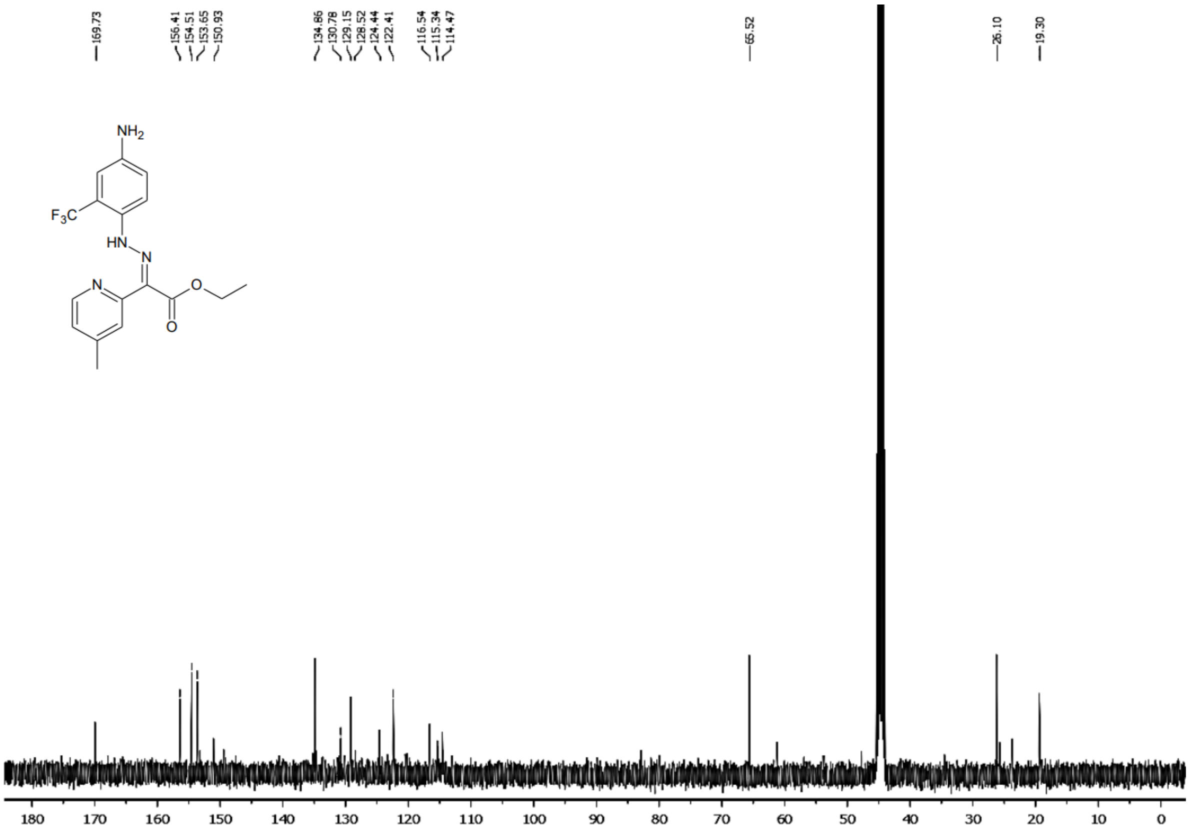

Figure S26. ^{13}C NMR spectrum of 4b in $(CD_3)_2SO$ at 298 K.

DFT calculations

DFT calculations were carried out using B3LYP hybrid functional combined with a 6-31G (d, p) basis set, CPCM (Acetonitrile). All calculations were performed using Orca 4.1.1 software.

Calculated compounds

Table S1. The list of compounds used for calculation of charge distribution. The structures are presented in Figure S27.

R_1	R_2
$-NO_2$	-H
-Cl	-H
-H	-H
$-CH_3$	-H
$-NH_2$	-H
-H	$-NO_2$
-H	-Cl
-H	$-CH_3$
-H	$-NH_2$
$-NH_2$	$-CH_3$
-Cl	$-CH_3$
$-OCH_3$	-Cl
$-NO_2$	-Cl

Regression analysis

$$\frac{[ZH^+]}{[ZH^+]+[E]} = \frac{c}{(1+d\cdot\exp(-f\cdot(pH-g)))} + 1 \quad (1)$$

Table S2. Regression parameters (c, d, f, g) for compounds 1a-1h, 2a-2b and 3a-3b fitted to eq 1 with Solver add-in in MS Excel.

compound	c	d	f	g
1a (4-NO2)	-0.920	0.318	4.289	0.780
1b (4-CN)	-1.220	0.707	1.792	1.222
1c (4-Br)	-0.937	3.094	3.037	2.097
1d (4-Cl)	-0.917	10.890	3.012	1.815
1e (4-H)	-0.898	7.418	4.699	2.841
1f (4-Me)	-0.917	5.322	4.122	3.416
1g (4-OMe)	-0.946	0.000	3.768	6.852
1h (4-NH2)	-0.907	3202.746	3.665	2.757
2a (4'-Cl)	-0.902	1.348	3.473	2.330
2b (4'-Me)	-0.918	6.714	5.366	4.064
3a (4,4'-Me)	-0.901	269.395	9.162	4.300
3b (4'-Me-4-OMe)	-0.911	0.645	4.785	5.370

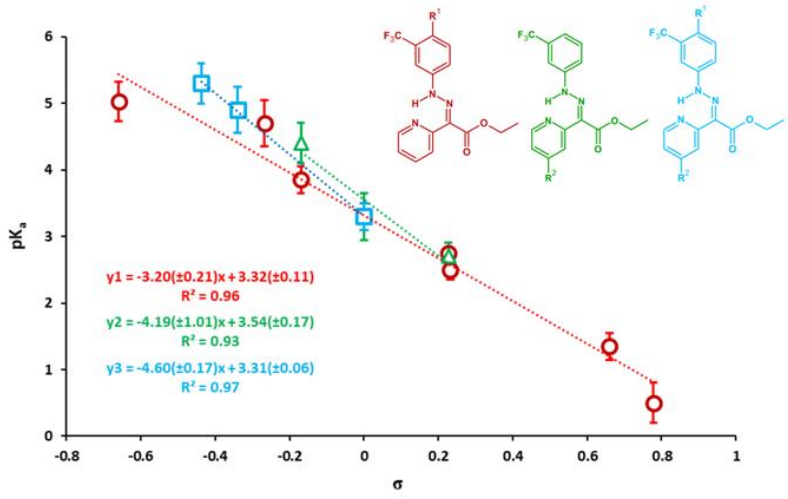

Figure S27. The relationship between the calculated pK_a values of compounds 1a-1h, 2a-2b and 3a-3b (based on eq 1 and Table S2) and the sum of Hammet constants of substituents R_1 and R_2.

UV-Vis spectra

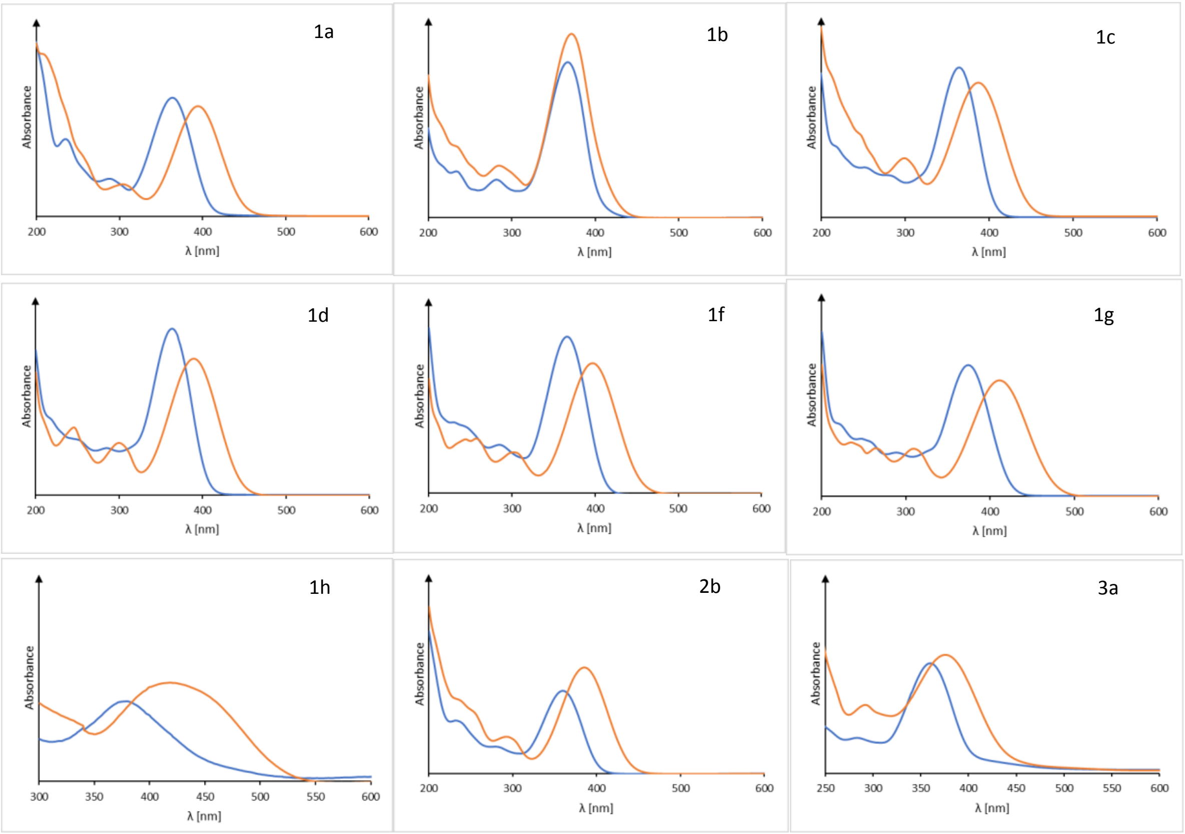

Figure S28. UV-Vis spectra of a 0.1 mM acetonitrile solution of compounds: 1a, 1b, 1c, 1d, 1f, 1g, 1h, 2b and 3a.

Field-inductive (σ_F) and resonance (σ_R) constants of selected substituents [1]

Table S3. σ_F and σ_R constants of selected substituents used for structure-property relationship calculations.

σ_R	σ_F	substituent
-0.48	0.09	$-NH_2$
-0.43	0.30	$-OCH_3$
-0.13	-0.01	$-CH_3$
0	0	-H
-0.16	0.43	-Cl
-0.16	0.49	-Br
0.18	0.54	-CN
0.16	0.64	$-NO_2$

[1] Hansch, C.; Leo, A.; Taft, R.W. A Survey of Hammett Substituent Constants and Resonance and Field Parameters. *Chem. Rev.* **1991**, *91*, 165–195, doi:10.1021/cr00002a004.

NMR properties comparison

Table S4. Comparison of ^{19}F NMR properties of molecular switch 4b and a fluorinated switch described in [2].

Compound	peak width [Hz]	T_1 [s]	T_2 [s]
4b (E)	8.8	1.540±111	1.557±069
4b (Z-H$^+$)	8.8	1.612±091	1.554±070
o-CF_3 switch (E) [2]	8.0	1.544±075	1.528±109
o-CF_3 switch (Z-H$^+$) [2]	8.0	1.611±089	1.551±082

[2] Janasik, D.; Jasiński, K.; Węglarz, W.P.; Nemec, I.; Jewula, P.; Krawczyk, T. Ratiometric pH-Responsive ^{19}F Magnetic Resonance Imaging Contrast Agents Based on Hydrazone Switches. *Anal. Chem.* **2022**, *94*, 3427–3431, doi:10.1021/acs.analchem.1c04978.
